# Improved Bioproduction of the Nylon 12 Monomer by Combining the Directed Evolution of P450 and Enhancing Heme Synthesis

**DOI:** 10.3390/molecules28041758

**Published:** 2023-02-13

**Authors:** Jiaming Yu, Jiawei Ge, Hongwei Yu, Lidan Ye

**Affiliations:** 1 Key Laboratory of Biomass Chemical Engineering (Education Ministry), College of Chemical and Biological Engineering, Zhejiang University, Hangzhou 310058, China; 2 Institute of Bioengineering, College of Chemical and Biological Engineering, Zhejiang University, Hangzhou 310058, China

**Keywords:** nylon 12, ω-AmDDA, P450, directed evolution, electron transfer, heme synthesis

## Abstract

The nylon 12 (PA12) monomer ω-aminododecanoic acid (ω-AmDDA) could be synthesized from lauric acid (DDA) through multi-enzyme cascade transformation using engineered *E. coli*, with the P450 catalyzing terminal hydroxylation of DDA as a rate-limiting enzyme. Its activity is jointly determined by the heme domain and the reductase domain. To obtain a P450 mutant with higher activity, directed evolution was conducted using a colorimetric high-throughput screening (HTS) system with DDA as the real substrate. After two rounds of directed evolution, a positive double-site mutant (R14R/D629G) with 90.3% higher activity was obtained. Molecular docking analysis, kinetic parameter determination and protein electrophoresis suggested the improved soluble expression of P450 resulting from the synonymous mutation near the N-terminus and the shortened distance of the electron transfer between FMN and FAD caused by D629G mutation as the major reasons for activity improvement. The significantly increased *k_cat_* and unchanged *K_m_* provided further evidence for the increase in electron transfer efficiency. Considering the important role of heme in P450, its supply was strengthened by the metabolic engineering of the heme synthesis pathway. By combining P450-directed evolution and enhancing heme synthesis, 2.02 ± 0.03 g/L of ω-AmDDA was produced from 10 mM DDA, with a yield of 93.6%.

## 1. Introduction

As an important engineering plastic, nylon 12 (PA12) has a number of advantages such as low water absorption, a high dimensional stability and a high temperature resistance and corrosion resistance and thus has wide applications in automobiles, electrical appliances, aerospace, etc. [1,2]. At present, the industrial production of PA12 mainly adopts the oxidation process, with butadiene as the raw material [3], but this process has problems such as the use of toxic and harmful raw materials, the need for a high reaction temperature, the dependence on nonrenewable petrochemical raw materials and the environmental stress caused. In contrast, the biosynthesis of the PA12 monomer from renewable resources is a green and sustainable process with mild reaction conditions, and it has thus emerged as a promising alternative.

**Figure 1:**
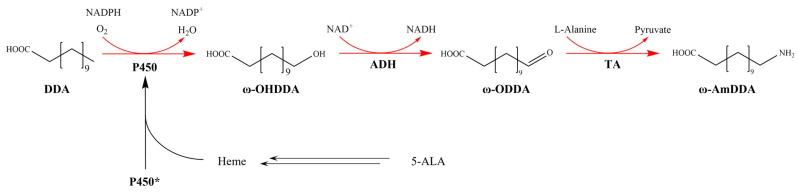
Biosynthetic pathway of ω-AmDDA. The double arrows indicate a multistep reaction. P450*, P450 without hydroxylation activity; DDA, dodecanoic acid; ω-OHDDA, 12-hydroxydodecanoic acid; ω-ODDA, ω-oxododecanoic acid; ω-AmDDA, ω-aminododecanoic acid; 5-ALA, 5-aminolevulinic acid.

Using methyl laurate as a raw material, the biosynthesis of 12-aminododecanoic acid methyl ester (ADAME) was realized through whole-cell catalysis with a yield of 12% (129 mg/L) [4]. The biotransformation of lauric acid (DDA) to ω-aminododecanoic acid (ω-AmDDA) was first reported in 2018 by using a mixture of two engineered strains, with a yield of 30% (93 mg/L) [5]. Recently, we constructed a cofactor self-efficient *E. coli* strain through the design of cofactor regeneration cycles and metabolic engineering of the chassis cell, which produced 1.04 g/L of ω-AmDDA from DDA at a yield of 96.5% [6]. In the biosynthetic pathway of ω-AmDDA (Figure 1), the P450 enzyme catalyzing the first step is well recognized as a rate-limiting step. If the catalytic activity of this enzyme could be improved, further improvement in the biosynthetic efficiency of the PA12 monomer could be expected.

The heme domain of P450 catalyzes the selective oxidation of inert hydrocarbon bonds through correct binding with the substrate. This reaction process relies on the coenzyme NAD(P)H and the complex electron transfer chain system. The shape and size of the substrate binding pocket, the efficiency of the electronic transfer chain system and the adequate supply of heme all contribute to the activity of P450. At present, most studies on P450 engineering focus on the heme domain [7,8,9]. For example, the G307A mutant of CYP153a from *Marinobacter aquaolei* enhanced the activity of the chimeric P450 enzyme (cyp153a-ncp) towards fatty acids by 2- to 20-fold. In recent years, there have also been a few reports on the engineering of the P450 reductase domain [8,10] and the interface between the heme domain and the reductase domain [8]. For example, the S120R/P165N/S453N mutant of CYP153a improved the electron transfer efficiency of redox partners to CYP153a, which increased its ω-hydroxylation activity towards oleic acid by 2.7-fold [8]. In comparison to rational or semi-rational design targeting selected residues, the directed evolution of the whole protein covering all three regions may generate mutants with higher activity.

For efficient directed evolution, an appropriate high-throughput screening (HTS) method is a necessity to facilitate the accurate selection of target mutants with desirable features from the huge random mutant library [11]. Diammonium 2,2′-azino-bis(3-ethylbenzothiazoline-6-sulfonate) (ABTS) colorimetry [12] has been used to screen P450 mutants with a high ω-hydroxylation activity of fatty acids. This method was developed based on the specific oxidation activity of a galactose oxidase mutant (GOase_M3-5_) towards terminal fatty acid hydroxylates, which can be coupled with the colorimetric measurement of H_2_O_2_ generated during this reaction. Therefore, this method may be suitable for the screening of P450 mutants with enhanced terminal hydroxylation activity of DDA. However, the Fe^2+/3+^ and D-glucose required for P450 catalysis and NADPH regeneration in the whole-cell reaction of DDA hydroxylation may interfere with the detection results of GOase_M3-5_ [13]. If the Fe^2+/3+^/Cu^2+^ ratio in the solution is too high, the Cu^2+^ binding of GOase_M3-5_ would be hindered, leading to a loss of activity. Meanwhile, the activity of GOase_M3-5_ towards D-glucose may generate false positive results. Therefore, this HTS method needs further improvement before it can be applied for P450-directed evolution in the whole-cell reaction system.

Moreover, the activity of catalytic C-H activation is mediated by the active P450 formed after the binding with heme. In the catalytic process, heme plays the key role of electron transfer, that is, it receives electrons from FMN and attacks the closest C-H bond on the substrate [14,15,16]. In engineered systems with P450 overexpression, the heme synthesized by the natural metabolism is often insufficient to match the massive amount of P450. The exogenous addition of 5-aminolevulinic acid (5-ALA) as a precursor of heme has been shown to improve the catalytic efficiency of cells overexpressing P450 enzymes [17,18]. However, *E. coli* generally has a low utilization rate of exogenous 5-ALA, strengthening the heme supply by metabolic engineering, which may therefore be a viable strategy for improving P450 performance. 

In our previously developed ω-AmDDA-producing *E. coli* strain [6], the chimeric P450 enzyme (cyp153a-ncp^G307A^) constructed by fusing the monooxygenase CYP153a^G307A^ mutant from *Marinobacter aquaolei* [9,19] and the reductase domain of P450 BM3 from *Bacillus megaterium* [20] was used. In order to further improve the ω-AmDDA bioproduction efficiency, in this study, the P450-catalyzed terminal hydroxylation was enhanced by both the directed evolution of cyp153a-ncp^G307A^ and the engineering of the heme synthesis pathway. A modified ABTS colorimetry-based HTS method was established and used for the directed evolution of the chimeric P450 construct, selecting mutants with enhanced catalytic activity. Meanwhile, the heme supply in the engineered strain was enhanced to provide sufficient active P450 by properly strengthening the heme synthesis pathway. Finally, the efficiency of this strategy was examined in ω-AmDDA bioproduction.

## 2. Results and Discussion

### 2.1. Establishment of the High-Throughput Screening Method

**Figure 2:**
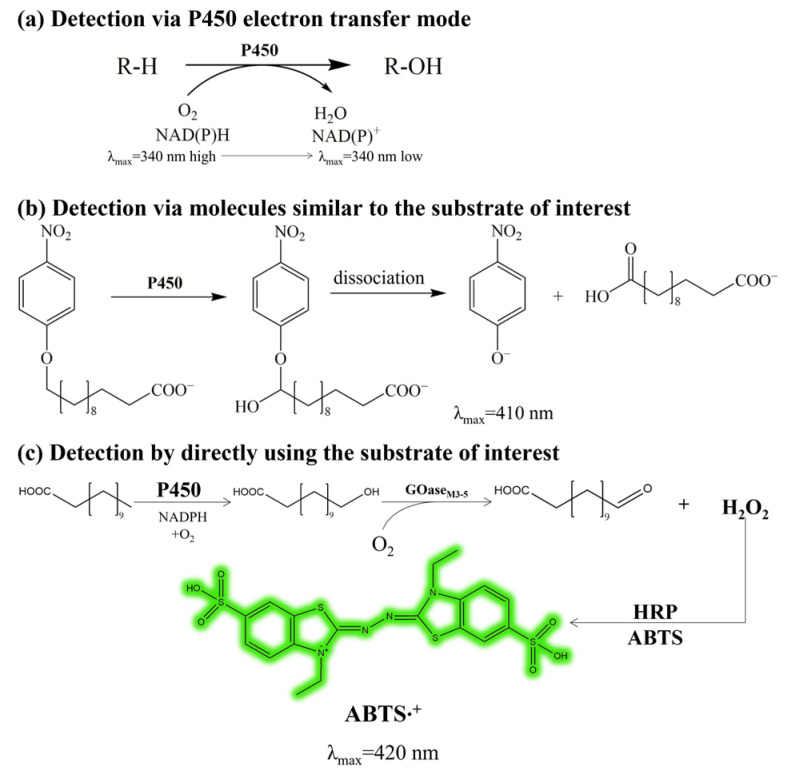
High-throughput screening (HTS) methods for P450. (**a**) Based on the P450 electron transfer mode, the P450 activity is assayed by measuring the NAD(P)H consumption. (**b**) Using ω-p-nitrophenoxycarboxylate acids (pNCA) as the artificial substrate, the terminal hydroxylation activity of P450 is assayed by measuring the yellow p-nitrophenoxy ions generated upon the dissociation of the product. (**c**) Using a mutant of galactose oxidase (GOase_M3-5_) with the specific oxidation activity of terminal fatty acid hydroxylates, the terminal hydroxylation activity of P450 could be assayed by quantifying the actual product ω-OHDDA via measuring the H_2_O_2_ generated during its oxidation to aldehyde by GOase_M3-5_. The colorimetric measurement of H_2_O_2_ is enabled by using horseradish peroxidase (HRP) and diammonium 2,2′-azino-bis(3-ethylbenzothiazoline-6-sulfonate) (ABTS). ABTS·^+^, the green–blue stable radical cationic chromophore formed by oxidation of ABTS.

The key to successful directed evolution lies in the availability of an efficient and reliable HTS method. The reported P450 enzyme activity assays are mostly based on NAD(P)H colorimetry (Figure 2a) [21], which is, however, unable to distinguish the regioselectivity of the enzyme. In ω-AmDDA biosynthesis, the highly selective terminal hydroxylation of DDA is a premise. Alternatively, p-nitrophenol colorimetry can be used to reflect the terminal hydroxylation activity of P450 using ω-p-nitrophenoxycarboxylate acids (pNCA) as artificial substrates (Figure 2b) [22]. However, the mutants selected with improved activity for the artificial substrates may not have the desirable performance for the target substrate. Recently, Weissenborn et al. [12] constructed an ABTS colorimetric method by coupling the galactose oxidase mutant GOase_M3-5_ capable of the specific oxidation of terminal fatty acid hydroxylates with horseradish peroxidase (HRP) (Figure 2c). In this HTS method, the site-specific catalytic activity of fatty acids can be measured and compared. Therefore, the GOase_M3-5_-based ABTS colorimetry seems to be applicable for the screening of P450 mutants with enhanced DDA terminal hydroxylation activity.

**Figure 3:**
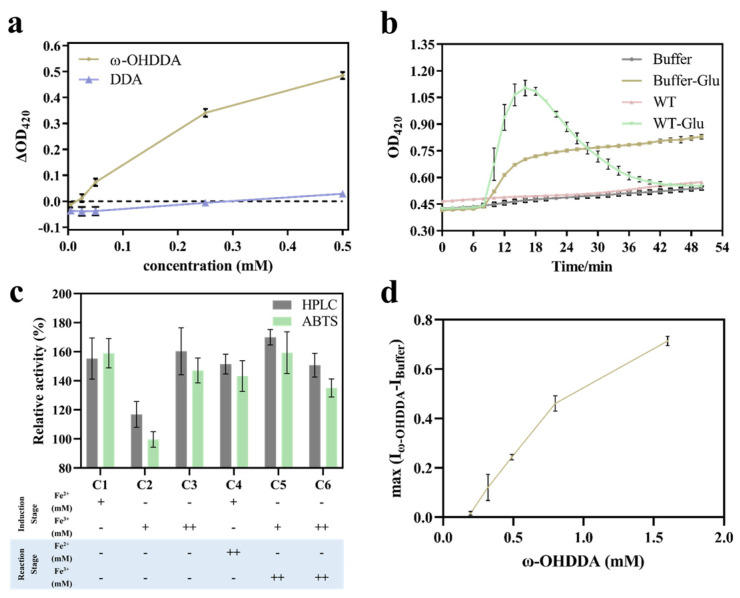
Establishment and optimization of the high-throughput screening method. (**a**) DDA and ω-OHDDA standard reagents were determined by ABTS colorimetry. ΔOD_420_, the maximum difference between the absorbance of the experimental group and the background at λ = 420 nm, namely, ΔOD_420_ = max (I_E_ − I_B_). (**b**) Results of ABTS colorimetry for the reaction systems with and without cyp153a-ncp (WT). The reaction was performed with a 50 g cell wet weight (cww)/L of WT cells in sodium phosphate buffer (100 mM, pH 8.0) with or without 1% (*w*/*v*) D-glucose. (**c**) Effect of Fe^2+/3+^ addition at different stages (protein induction stage and whole-cell reaction stage) on the accuracy of the ABTS colorimetry method, as shown by its consistency with the HPLC determination results. C1-C6 stands for conditions 1–6 with different iron addition strategies. The y-axis shows the relative activity of B1-1 (E-M1-3) containing M1 (cyp153a-ncp^G307A^), as compared to B1-1 (E-3) containing WT (cyp153a-ncp). +, 0.15 mM; ++, 0.5 mM. (**d**) Correlation between the maximum difference value between the absorbance value of the product and the background and the product concentration. I, absorbance value at 420 nm; max (), the maximum value of a set. The reaction system contained sodium phosphate buffer (100 mM, pH 7.5), 25 μL whole-cell reaction product, 27.5% (*v*/*v*) GOase_M3-5_, 22.5 U/mL HRP, CuSO_4_ 0.05 mM and ABTS 0.4 mM.

To validate the feasibility of this HTS method for the directed evolution of P450 towards higher DDA terminal hydroxylation activity, the correlation between the absorbance and ω-OHDDA amount was first examined (Figure 3a). Subsequently, cyp153a-ncp (WT) and cyp153a-ncp^G307A^ (M1), with a known activity difference, were tested as the low and high enzyme activity conditions, respectively, to further verify the feasibility of this method in the whole-cell catalysis system. Considering the Fe^2+/3+^ and Cu^2+^ dependence of cyp153a-ncp and GOase_M3-5_, respectively, the effect of Fe^2+/3+^ addition on the activity of GOase_M3-5_ and, thus, on the results of ABTS colorimetry was investigated (Figure 3c). The addition of 0.15 mM Fe^2+^ at the protein induction stage was found to be the best, with good consistency with the HPLC results. In addition, the whole-cell reaction system contained 1% (*w*/*v*) D-glucose for the D-glucose dehydrogenase (GDH)-mediated NADPH regeneration to support the hydroxylation reaction, while GOase_M3-5_, as a glucose oxidase, has D-glucose oxidation activity and could generate H_2_O_2_ in this process [13], which may interfere with the colorimetric reaction and lead to false positive results. The exclusion of D-glucose from the reaction system led to limited ω-OHDDA formation, which was below the detection limit of this colorimetric method (Figure 3b). To avoid the interference of D-glucose, we tried to replace the GDH system with other NADPH regeneration systems, including ICD [23], GDHA [24], FDH [25], FDH-PNTAB [26,27] and FDH-STHA [26,27]. However, all those NADPH regeneration systems were not as effective as the GDH system (Supplementary Materials: Figure S1).

Fortunately, GOase_M3-5_ was much more sensitive to ω-OHDDA than D-glucose (Figure 3b). Using the absorbance of the buffer containing D-glucose as the background value, the result calculated by subtracting the background value from the absorbance value of the reaction product (I = max (I_ω-OHDDA_ − I_Buffer_)) had a linear relationship with the concentration of ω-OHDDA in the range of 0.2–0.8 mM (Figure 3d). In this way, the false positive result caused by D-glucose in the system could be avoided. This modified HTS method was named ABTS 2.0 colorimetry. 

### 2.2. Directed Evolution of cyp153a-ncp^G307A^ (M1)

**Figure 4:**
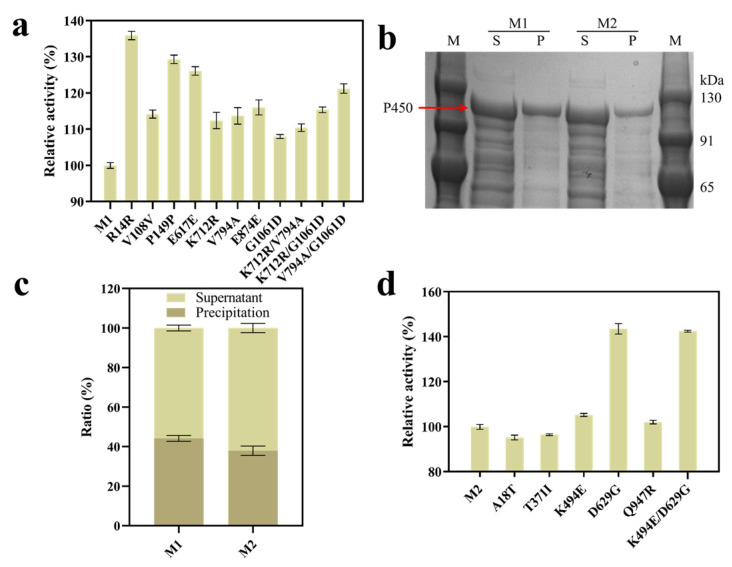
Combinatorial mutagenesis of hot spots identified in directed evolution. (**a**) With M1 as the parent, single-point mutants and combined mutants were constructed according to the results of the first round of screening, and each mutant contained the G307A mutation. The enzyme activity of M1 was set as 100%. (**b**) The expression of M1 and M2 (cyp153a-ncp^G307A/R14R^). Lane M, marker; lane S, supernatant; lane P, precipitation. (**c**) Expression analysis of P450 mutants. SDS-PAGE protein electrophoresis results were analyzed by ImageJ software to calculate the proportions of P450 in the supernatant and the precipitation of M1 and M2, respectively. (**d**) With M2 as the parent, single-point mutants and combined mutants were constructed according to the results of the second round of screening, and each mutant contained the G307A/R14R mutation. The enzyme activity of M2 was set as 100%.

The P450 mutant library was constructed with M1 as the parent by using error-prone PCR (epPCR), and the mutation rate was controlled to 1–2 bp per kb. The mutant gene fragments were cloned into the expression plasmid E-M1-3 [6], and 15 mM DDA was used as the substrate for the HTS of the library. The mutants with increased ω-OHDDA production were preliminarily screened by ABTS 2.0 colorimetry and confirmed by HPLC. In order to ensure the accuracy of the screening, the reaction solution of the whole-cell biotransformation was diluted to let the ω-OHDDA concentration fall in the range of 0.2–0.8 mM before the measurement with ABTS 2.0 colorimetry. Among 1200 clones, 5 mutants with improved activity were obtained (Table 1). The mutant with R14R synonymous mutation showed a 35.9% higher activity than M1 (Figure 4a), and the yield of ω-OHDDA from 15 mM DDA reached 71.1% within 4 h. Because the R14 site is located at the N-terminus of P450 and the change in the codon preference near the N-terminus was reported to have a great impact on protein expression [28,29], the SDS-PAGE analysis of the mutants was conducted. The result showed an improvement in the soluble expression of the protein after the R14R synonymous mutation (Figure 4b,c), which was possibly due to the slight increase in the codon preference (from 0.36 to 0.37) and the moderate reduction in the translation rate (from 55,270 to 32,498), as calculated by the ribosome binding site (RBS) calculator [30,31] [https://salislab.net/software/ (accessed on 10 January 2023)].

In the second round, cyp153a-ncp^G307A/R14R^ (M2) was used as the parent, and 1 mutant with improved activity was screened out from 800 clones (Table 1). The D629G mutation delivered 41.3% activity improvement (Figure 4d), and the yield of ω-OHDDA from 15 mM DDA reached 85.8% within 4 h. Because the D629 site was located in the FMN binding domain of the P450 reductase domain, we speculated that the enhancement of P450 activity in mutant M3 (cyp153a-ncp^G307A/R14R/D629G^) might result from the improvement of electron transfer efficiency. The determination of the kinetic constant confirmed that the increase in catalytic activity was due to the increase in turnover numbers rather than the increase in substrate affinity (Table 2). This result highlights the important role of electron transfer in P450-catalyzed reactions, which is in accordance with previous reports [8,10].

### 2.3. Molecular Simulation Analysis

**Figure 5:**
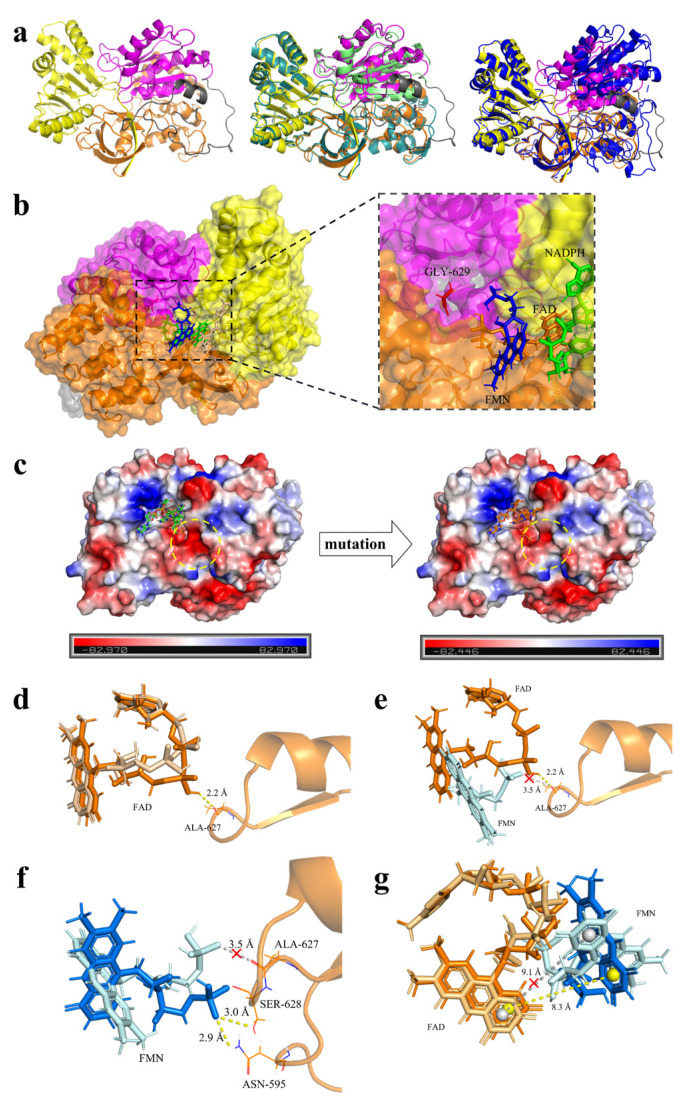
Molecular modeling and docking analysis of M1 and M3. (**a**) Homologous modeling was conducted for the reductase domain (NCP) of P450. From left to right: the homology modeling of NCP, the comparison results between NCP, 1bvy (PDB code: 1bvy [14], green) and 4dqk (PDB code: 4dqk [32], cyan), the comparison results between NCP and 1amo (PDB code: 1amo [33], blue). The structural domains of NCP are represented by different colors: the NAP domain in yellow, the FMN domain in magenta, the FAD domain in orange, the random coil in gray. (**b**) The docking results of M3 with FAD, NADPH and FMN molecules. The small molecules are represented by different colors: NADPH in green, FMN in blue, FAD in orange. G629 is represented by red. (**c**) Protein surface electrostatic potential display of M1 (left) and M3 (right). (**d**–**g**) Local display of docking results of FAD and FMN molecules with M1 and M3. FAD formed a new hydrogen bond with A627 (**d**) to replace the original long hydrogen bond (**e**). FMN forms new hydrogen bonds with N595 and S628, respectively (**f**). The distance between FMN and FAD was shortened (**g**). The small molecules of the M1 docking results are presented in light colors, and those of M3 are presented in dark colors. Hydrogen bonds at key positions of M1 and M3 are indicated by white dotted lines and yellow dotted lines, respectively. The red “x” indicates that these hydrogen bonds are broken in M3.

In order to further explore the molecular mechanism of the enhanced catalytic activity in the D629G-containing mutant M3, molecular simulation analysis was carried out. Considering that the D629 site is located in the P450 reductase domain, the P450 reductase domain model (including the FAD, NAP and FMN binding domains) was first constructed through homologous modeling. In this process, we paid special attention to the arrangement of domains based on the direction of electron transmission: NADPH → FAD → FMN (Figure 5a). The FMN binding domain referred to the 1bvy model (PDB code: 1bvy [14]), with a sequence homology of 100%, the FAD and NAP binding domains referred to the 4dqk model (PDB code: 4dqk [32]), with a sequence homology of 100%, and the splicing of the three modules referred to the 1amo model (PDB code: 1amo [33]), with the same domains and similar structures (Figure 5a). The evaluation results using MolProbity [34] [http://molprobity.biochem.duke.edu/ (accessed on 28 November 2022)] and SAVES [35] [https://saves.mbi.ucla.edu/ (accessed on 28 November 2022)] demonstrated the high accuracy of this model. The evaluation results using MolProbity showed that 93.2% (549/589) of all residues were in favored (98%) regions, and 97.1% (572/589) of all residues were in allowed (>99.8%) regions (Figure S2), the evaluation results using VERIFY3D showed that this model has 89.17% of the residues with an averaged 3D-1D score >= 0.2, and the evaluation results using the Ramachandran plot showed that this model has 89.7% (468/522) of the residues in most favored (>90%) regions (Figure S3). The docking results of FAD, NADPH and FMN molecules with this model showed that the D629G mutation site is located in the FMN binding domain and near the cofactor binding pocket (Figure 5b). When the acidic aspartate was substituted by the neutral glycine, the surface electrostatic potential of the cofactor binding pocket was increased (Figure 5c), which was favorable for the negatively charged phosphate group of FAD in approaching and competing with the O1P of FMN for A627. The formation of a new hydrogen bond with a shorter distance (2.2 Å) to replace the original long hydrogen bond (3.5 Å) (Figure 5d,e) allowed for the rotation of FMN to a certain angle for the formation of new hydrogen bonds with N595 (2.9 Å) and S628 (3.0 Å), respectively (Figure 5f). Meanwhile, the distance between the rotated FMN and FAD was shortened from 9.1 Å to 8.3 Å (Figure 5g), and the shorter distance was conducive to the transfer of electrons from FAD to FMN [10,36,37], thereby improving the catalytic efficiency of P450. In addition, the performance of the mutant M3 was investigated for other fatty acids of different lengths, finding that the enzyme activities were improved for all of the substrates tested (Table 3), while retaining the specificity of terminal hydroxylation (Figures S4 and S5). This result showed that the mutant generated by the increasing electron transfer efficiency improved the catalytic performance in a substrate-independent manner, implying wide applications of such mutants. This also provides a reference for the modification of P450 and other enzymes with similar electron transfer domains.

### 2.4. Enhancement of the Heme Synthesis Pathway

**Figure 6:**
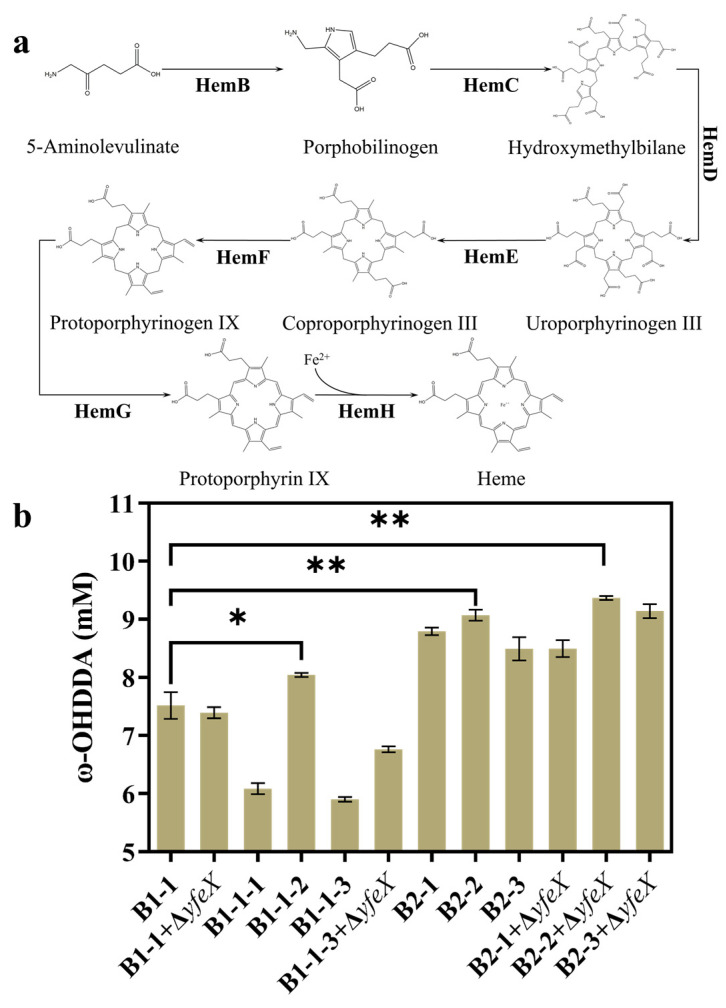
Enhancement of heme synthesis in *E. coli*. (**a**) Heme synthesis pathway of *E. coli*. (**b**) ω-OHDDA production in whole-cell systems modified with different strategies. B1-1 without the modification of heme synthesis was used as the control strain. Statistical analysis was performed by an unpaired two-tailed t-test. * 0.01 < *p* < 0.05; ** *p* < 0.01. B2-1, B2-2 and B2-3 used promoters with different strengths (P*_lac_*, P*_tac_* and P*_T7_*, respectively) for *hemB* overexpression. The E-M1-3 plasmid was expressed in the control group and in each experimental group.

Only when the heme domain of P450 binds to heme can the enzyme catalyze C-H activation [14,15,16]. Therefore, the adequate supply of heme in cells is essential to increasing the proportion of active P450. However, the heme synthesized by *E. coli* is limited (Figure 6a). In order to improve its synthesis, many optimization strategies have been reported. Weng et al. [38] overexpressed the *hemA*, *hemB*, *hemC*, *hemD*, *hemE*, *hemF*, *hemG*, *hemH* and *hemL* genes using the pUC19-hemAL, pACYCDuet-2-hemBCDE and pRSFDuet-2-hemFGH plasmids and increased the heme synthesis to 0.82 mg/L in *E. coli*. Zhao et al. [18] overexpressed the *hemA* and *hemL* genes on the pCDF-hemAL, *hemB*, *hemC* and *hemD* genes on the pRSF-hemBCD, *hemE*, *hemF*, *hemG* and *hemH* genes on pET-hemEFGH and knocked out the lactic acid- and acetate-forming genes (*ldhA* and *pta*) and the *yfeX* gene to enhance the supply of 5-ALA precursors and prevent heme degradation, respectively, which, together, increased the heme synthesis to 6.6 ± 0.2 mg/L. It has also been reported that optimizing the expression levels of the HemB, HemG and HemH enzymes alone promoted the transformation from 5-ALA to heme [39]. Ge et al. [17] found that the overexpression of the *hemB* gene alone led to a slight increase in the accumulation of 5-ALA, while the co-overexpression of the *hemB*, *hemG* and *hemH* genes had no significant impact on the production of 5-ALA. Significant heme accumulation was observed in both cases. Based on these reports, we tested three pathway engineering strategies and compared their efficiency: 1. Overexpressing *hemB*, *hemC*, *hemD* and *hemE* on a low-copy plasmid (pAC-hemBCDE), and overexpressing *hemF*, *hemG* and *hemH* on a high-copy plasmid (pRS-hemFGH) (B1-1-1); 2. Overexpressing hemB on a low-copy plasmid (pAC-hemB) (B1-1-2); 3. Overexpressing *hemB*, *hemC* and *hemD* on a high-copy plasmid (pRS-hemBCD), overexpressing *hemE*, *hemF*, *hemG* and *hemH* on a low-copy plasmid (pAC-hemEFGH) (B1-1-3) and deleting the *yfeX* gene (B1-1-3+Δ*yfeX*).

It turned out that strategy 2, namely, overexpressing *hemB* on low-copy plasmid, led to the best result, which increased the ω-OHDDA yield of B1-1-2 by 7.0% (Figure 6b). Then, we compared the effect of integrative HemB expression driven by promoters of different strengths and found medium-intensity overexpression to be the best, which increased the ω-OHDDA yield of B2-2 by 20.7% (Figure 6b). In addition, it was found that the single deletion of *yfeX* had little effect on the product yield in B1-1, while its deletion in B1-1-3 with a background of strengthened heme synthesis improved the product yield. This result was suggested only when heme was massively synthesized, blocking its degradation-exerted positive effect. Therefore, we tentatively deleted *yfeX* from strain B1-1-2 with *hemB* upregulation and found a 24.6% increase in the yield of ω-OHDDA. These results indicated that the enhancement of the intracellular heme supply could effectively increase the proportion of active P450 (Figure S6), thereby increasing the production of related products.

### 2.5. Construction of the ω-AmDDA Synthesis Pathway

**Figure 7:**
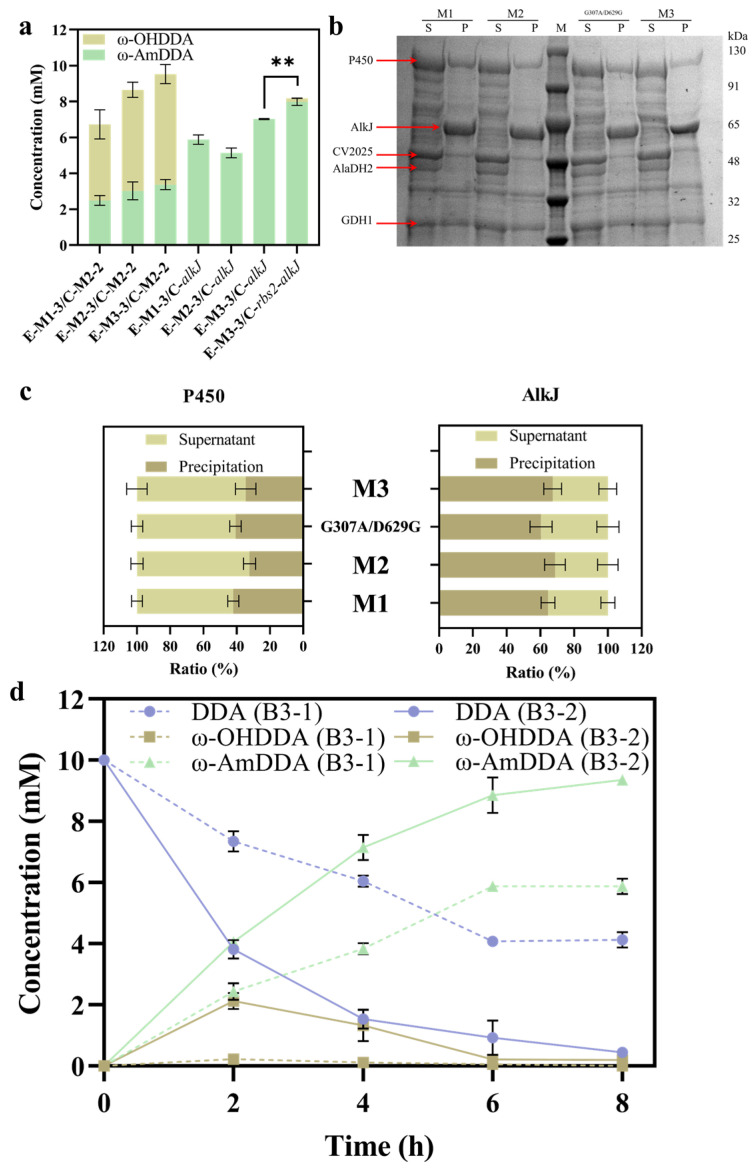
Construction of the ω-AmDDA synthesis pathway in *E. coli*. (**a**) ω-AmDDA production in the P1-1 strain expressing M1, M2 and M3 together with BsADH or AlkJ. The P450 in E-M1-3 was M1, while those in E-M2-3 and E-M3-3 were the P450 mutants M2 and M3, respectively. Statistical analysis was performed by an unpaired two-tailed t-test. ** *p* < 0.01. (**b**) Expression of P450, AlkJ, CV2025, AlaDH2 and GDH1. Lane M, marker; lane S, supernatant; lane P, precipitation; AlkJ, alcohol dehydrogenase. (**c**) Expression analysis of P450 mutants and AlkJ. SDS-PAGE protein electrophoresis results were analyzed by ImageJ software to calculate the proportions of P450 and AlkJ in the supernatant and the precipitation of M1, M2, G307A/D629G (cyp153a-ncp^G307A/D629G^) and M3, respectively. (**d**) Biotransformation of DDA to ω-AmDDA by B3-1 (P1-1 (E-M1-3/C-*alkJ*)) and B3-2 (P1-1, Δ*yfeX*-*T10*::*P_tac_-hemB* (E-M3-3/C-*rbs2*-*alkJ*)). The reaction was performed with 50 g cww/L of resting cells in sodium phosphate buffer (100 mM, pH 8.0) containing 1% (*w*/*v*) D-glucose, NH_3_·H_2_O/NH_4_Cl (200 mM, NH_3_·H_2_O:NH_4_Cl = 1:10) and 10.0 mM DDA (2% DMSO). The temperature was maintained at 30°C, the agitation speed was maintained at 220 rpm, the pH was maintained at 7.5–8.0 and the concentration of D-glucose was maintained at 0.5–1% (*w*/*v*) throughout the biotransformation process. All biotransformation reactions were performed in triplicate, and error bars represent standard deviations.

By replacing the chimeric P450 (M1) in the previously constructed *E. coli* strain P1-1 (E-M1-3/C-M2-2) [6] with the P450 mutants M2 (cyp153a-ncp^G307A/R14R^) and M3 (cyp153a-ncp^G307A/R14R/D629G^) obtained from directed evolution, the *E. coli* strains P1-1 (E-M2-3/C-M2-2) and P1-1 (E-M3-3/C-M2-2) were generated. As compared to the previous strain, the ω-AmDDA production was increased by 21.5% and 35.6%, respectively (Figure 7a). Meanwhile, the obvious accumulation of the intermediate ω-OHDDA was observed, suggesting that the conversion rather than the formation of ω-OHDDA became the rate-limiting step, which may be related to the massive consumption of NADPH by the enhanced P450 catalytic reaction, leading to the decreased pool of NAD^+^/NADP^+^ in the cells [40,41]. The alcohol dehydrogenase BsADH, responsible for further catalyzing ω-OHDDA to ω-ODDA, was NAD^+^-dependent [42], so the lower NAD(H) pool may limit the activity of this enzyme, thus leading to the accumulation of ω-OHDDA. For this reason, we replaced BsADH with the FAD-dependent alcohol dehydrogenase (AlkJ) [5,43] from *Pseudomonas oleovorans*, thus avoiding the accumulation of the intermediate and further improving the output of ω-AmDDA (Figure 7a). However, we found that the introduction of the P450 mutant had a negative impact on the expression of AlkJ (Figure 7b,c), which might be related to the increase in the metabolic burden caused by the improved P450 expression. Such phenomenon was also observed in other studies on multi-enzyme cascade biotransformation in *E. coli* [6,44]. In order to restore the expression level of AlkJ, a stronger RBS2 (TCAATAGCCTTGACTAAGGAGGTAACT), as predicted by the RBS calculator [30,31] [https://salislab.net/software/ (accessed on 17 August 2022)], was introduced in front of AlkJ (C-*rbs2*-*alkJ*), which solved the expression problem of AlkJ and increased the output of ω-AmDDA to 7.98 ± 0.19 mM (Figure 7a).

In order to further improve the production of ω-AmDDA, we introduced the abovementioned plasmid into the P1-1, Δ*yfeX*-T10::*P_tac_-hemB-T_1_* strain to construct the B3-2 (P1-1, Δ*yfeX*-T10::*P_tac_-hemB-T_1_* (E-M3-3/C-*rbs2*-*alkJ*)) strain. Compared with B3-1 (P1-1 (E-M1-3/C-*alkJ*)), obviously more ω-OHDDA was produced within the first 2 h, while it was almost completely converted to ω-AmDDA after 6 h of the reaction (Figure 7d). Finally, the yield of ω-AmDDA from 10 mM DDA reached 93.6% within 8 h, which was 17.3% higher than that of P1-1 (E-M3-3/C-*rbs2*-*alkJ*) with the P450 mutant M3 but without the modification of the heme synthetic pathway (Figure 7a), 59.4% higher than B3-1 with the original P450 variant M1 and without the modification of the heme synthetic pathway (Figure 7d) and 227.0% higher than the P1-1 (E-M1-3/C-M2-2) constructed in the previous study [6] (Figure S7). These results demonstrated the combination of P450-directed evolution and heme synthesis engineering to be an efficient strategy for promoting ω-AmDDA production.

## 3. Materials and Methods

### 3.1. Strains and Plasmids

The plasmids used in this study are listed in Table 4. The primers used for constructing plasmids are listed in Table S1. *E. coli* BL21(DE3), Δ*fadD*::*P_lacUV5_*-*alkL* (B1-1, laboratory preservation) [6] was used for gene cloning and expression. B1-1, Δ*fadD*::*P_T7_-yaaDE* (P1-1, laboratory preservation) [6] was used for ω-AmDDA synthesis. The plasmid pETDuet-1-*rbs3*-*cyp153a-ncp^G307A^*-*gdh1* (E-M1-3, laboratory preservation) [6] was used for P450 transformation and expression, which contained the complete encoding sequences of cyp153a-ncp^G307A^ and GDH1 (for NADPH regeneration). The plasmid pCDFDuet-1-*BsADH^C257L^*-*cv2025*-*aladh2* (C-M2-2, laboratory preservation) [6] was used to express the enzyme for converting ω-OHDDA into ω-AmDDA. The galactose oxidase mutant (GOase_M3-5_) [13] was synthesized by Generay Biotech Co., Ltd. (Shanghai, China) and expressed on pET-30a(+)-M3-5. The NADPH-preferred formate dehydrogenase gene (*fdh1*) [25] from *Burkholderia stabilis 15516*, the NADH-dependent formate dehydrogenase gene (*fdh2*) [26] from *Mycolicibacterium vaccae* and the FAD-dependent alcohol dehydrogenase gene (*alkJ*) [5,43] from *Pseudomonas oleovorans* were codon-optimized and synthesized by Generay Biotech Co., Ltd. (Shanghai, China). All the other genes (*icd*, *gdhA*, *pntAB*, *sthA*, *hemB*, *hemC*, *hemD*, *hemE*, *hemF*, *hemG* and *hemH*) were amplified from the genomic DNA of *E. coli* K12 (MG1655). The genome editing of *E. coli* was conducted by the transposon method using tools kindly provided by Professor Sheng Yang from the Shanghai Institute for Biological Sciences [45]. The three expression plasmids (pCDFDuet-1, pRSFDuet-1 and pACYCDuet-1) used in this study were kindly provided by Professor Zhi Li from the National University of Singapore [46].

To construct the plasmids pAC-*hemB*, pAC-*hemE* and pAC-*hemH*, the *hemB*, *hemE* and *hemH* genes were amplified using the primers hemB-2-F/hemB-2-R, hemE-1-F/hemE-1-R and hemH-2-F/hemH-1-R, respectively, and the plasmid pACYCDuet-1 was amplified using pACYC-2-F/pACYC-2-R. The amplification products were connected by the Gibson assembly method [47]. To construct the plasmid pAC-hemBCDE, the *hemB*, *hemC* and *hemD* genes were amplified using the primers hemB-1-F/hemB-1-R, hemC-1-F/hemC-1-R and hemD-1-F/hemD-1-R, respectively, and the plasmid pAC-*hemE* was amplified using pACYC-1-F/pACYC-1-R, followed by the Gibson assembly of the amplification products. To construct the plasmid pAC-hemEFGH, the *hemE*, *hemF* and *hemG* genes were amplified using the primers hemE-2-F/hemE-2-R, hemF-2-F/hemF-2-R and hemG-2-F/hemG-2-R, respectively, and the plasmid pAC-hemH was amplified using pACYC-4-F/pACYC-3-R, followed by the Gibson assembly of the amplification products. The plasmids pRS-*hemB*, pRS-*hemF*, pRS-hemBCD and pRS-hemFGH were constructed by the same method. The plasmids pETDuet-1-*rbs3*-*cyp153a*-*ncp^G307A^*-*gdhA* (E-M1-3-*gdhA*), pETDuet-1-*rbs3*-*cyp153a*-*ncp^G307A^*-*icd* (E-M1-3-*icd*), pETDuet-1-*rbs3*-*cyp153a*-*ncp^G307A^*-*fdh1* (E-M1-3-*fdh1*), pCD-*pntAB*, pCD-*sthA*, pCD-*pntAB*-*fdh2* and pCD-*sthA*-*fdh2* were constructed by digestion and ligation. The plasmids C-*alkJ* and C-*rbs2*-*alkJ* were constructed by Golden Gate assembly [48].

### 3.2. Chemicals and Enzymes

ω-AmDDA was purchased from TCI (Shanghai, China). DDA and ω-OHDDA were purchased from Adamas-beta Ltd. (Shanghai, China). ABTS and other chemicals were purchased from Sangon Biotech Ltd. (Shanghai, China). Easy Taq DNA polymerase, PrimeSTAR^®^ HS DNA Polymerase, restriction endonucleases and T4 DNA ligase were purchased from TAKARA Ltd. (Dalian, China). HRP was purchased from Sangon Biotech Ltd. (Shanghai, China). Oligonucleotides were synthesized by Sangon Biotech Ltd. (Shanghai, China) and Tsingke Biotechnology Co., Ltd. (Beijing, China).

### 3.3. Expression, Purification and SDS-PAGE Analysis of Galactose Oxidase

The BL21+M3-5 strain was cultured overnight in 5 mL Luria-Bertani broth (LB, containing 50 mg/L Kanamycin) at 37 °C and 220 rpm as a seed culture, which was transferred to fresh LB (containing 50 mg/L Kana) with an inoculation volume of 5% (*v*/*v*) for further cultivation at 37 °C and 220 rpm until the OD_600_ reached 0.6. For the protein induction, 0.1 mM IPTG and 0.5 mM CuSO_4_ were added and cultured at 20 °C and 180 rpm for 10–16 h. The cells were collected by centrifugation and then washed and resuspended to a 50 g cell wet weight (cww)/L with sodium phosphate buffer (100 mM, pH 7.5) for ultrasonic cell disruption (200 W~400 W power, 90 cycles of 4 s fragmentations and 4 s intervals). After centrifugation, the GOase_M3-5_ crude enzyme was obtained as the supernatant, which was loaded onto 10% sodium dodecyl sulfate polyacrylamide gel for electrophoresis analysis and used for ABTS 2.0 colorimetry.

### 3.4. Cloning, Expression and Purification of P450

The construction of the P450 mutant library was carried out using epPCR. The plasmid E-M1-3 carrying the *cyp153a-ncp^G307A^* gene was used as the template to generate a random library through epPCR. Mutagenesis was performed on the full sequence of *cyp153a-ncp^G307A^*, using the CYPCPR-Gibson-F/CYPCPR-Gibson-R primers (Table S1). The epPCR reaction mixture contained 0.1~0.2 ng plasmid template, 0.2 mM dNTPs, 0.1 mM MnCl_2_ and 5 U Taq DNA polymerase. The mutated *cyp153a-ncp^G307A^* was ligated to E-M1-3 [6] through the Gibson assembly method to replace *cyp153a-ncp^G307A^* on the original plasmid.

The cloning of His-Tagged P450: His-Tag was integrated into the N-terminus and C-terminus of cyp153a-ncp^G307A^ by the Golden Gate method. The PCR primers used were TP450-BsaI-GCCA-F/TP450-BsaI-TACC-R and TP450-BsaI-ACTG-F/TP450-BsaI-AGGC-R, respectively (Table S1). The fragments containing His-Tag were obtained by primer annealing, and the primers used were NHis-BsaI-F/NHis-BsaI-R and Chis-BsaI-F/Chis-BsaI-R (Table S1).

Expression of P450: The B1-1 [6] strain was used as the expression host. The preliminary screening was carried out in 96 deep-well plates. Single colonies were inoculated to 300 μL LB (containing 100 mg/L Amp) and incubated at 37 °C and 220 rpm for more than 10 h, and then 100 μL fresh seed solution was transferred to 24-well plates containing 2 mL Terrific broth (TB, containing 100 mg/L Amp) and cultured at 37 °C and 220 rpm until the OD_600_ reached 0.8~1. For the protein induction, 0.1 mM IPTG, 1% (*w*/*w*) trace element stock solution (containing 0.15 M Fe^2+^) [49], 0.5 mM 5-ALA and 65 mg/L VB1 were added. After culturing at 24.5 °C and 180 rpm for 10–16 h, the cells were collected for the whole-cell biotransformation and purification of P450.

The purification of His-tagged P450: The crude enzyme of P450 was mixed with Binding Buffer (200 mL/L sodium phosphate buffer (100 mM, pH 7.5), 29.2 g/L NaCl and 1.36 g/L imidazole) at 1:1 and passed through the Ni-NTA column [affinity, agarose resin, Thermo Scientific™ (Waltham, MA, USA)], followed by washing with Wash Buffer (200 mL/L sodium phosphate buffer (100 mM, pH 7.5), 29.2 g/L NaCl and 3.4 g/L imidazole). P450 was eluted using Elution Buffer (200 mL/L sodium phosphate buffer (100 mM, pH 7.5), 29.2 g/L NaCl and 34 g/L imidazole). Eluents containing His-Tagged P450 (120 kDa) were pooled, and the buffer was exchanged with sodium phosphate (100 mM, pH 7.5) by an Amicon^®^ Ultra-30 centrifugal filter [Merck Millipore Ltd. (Darmstadt, Germany)] with a molecular cut-off value of 30 kDa. The concentration of the purified P450 was determined with a BCA kit [Sangon Biotech Ltd. (Shanghai, China)] and CO spectra [50,51].

### 3.5. Whole-Cell Biotransformation

The cells were washed with sodium phosphate buffer (100 mM, pH 7.5). The reaction was performed with 50 g cww/L of resting cells in sodium phosphate buffer (100 mM, pH 8.0) containing 1% (*w*/*v*) D-glucose, NH_3_·H_2_O/NH_4_Cl (200 mM, NH_3_·H_2_O:NH_4_Cl = 1:10) and 15.0 mM DDA (2% DMSO) for 2–8 h. The temperature was maintained at 30 °C, the agitation speed was maintained at 220 rpm, the pH was maintained at 7.5–8.0 and the concentration of D-glucose was maintained at 0.5–1% (*w*/*v*) throughout the biotransformation process. For rescreening, 1% (*w*/*w*) trace element stock solution (containing 0.5 M Fe^2+^) [49] was added during the reaction. All biotransformation reactions were performed in triplicate.

### 3.6. High-Throughput Screening of P450

ABTS 2.0 colorimetry determination was completed in 96-well plates with 200 μL of the reaction system: 25 μL whole-cell biotransformation solution, 27.5% (*v*/*v*) crude enzyme solution of GOase_M3-5_, 4.5 U HRP, CuSO_4_ 0.05 mM, ABTS 0.4 mM in sodium phosphate buffer (100 mM, pH 7.5). The Epoch 2 microplate reader (BioTek Instruments, Inc., Winooski, VT, USA) was used to measure the absorbance value at 420 nm and 30 °C every 1–4 min.

### 3.7. HPLC and GC/MS Analysis

For the HPLC analysis, the reaction solution of whole-cell biotransformation was mixed with the same volume of acetonitrile, and trifluoroacetic acid (TFA) was added to terminate the reaction. The contents of ω-OHDDA and ω-AmDDA were detected by HPLC using Phenomenex SHI-MADZU LC-20 AT equipped with a C8(2) column (Aschaffenburg, Germany). The mobile phases were water containing 0.1% (*w*/*w*) TFA (phases A) and methanol containing 0.1% (*w*/*w*) TFA (phases B), respectively. A gradient elution program was used: 0–2 min 70% A/30% B, 2–5 min 40% A/60% B, 5–14 min 15% A/85% B, 14–25 min 15% A/85% B, 25–32 min 2% A/98% B, 32–35 min 70% A/30% B. The detector was the ELSD Detector (Alltech 3300) (temperature 65 °C, nitrogen flow rate 1.5 L/min), the injection volume was 20 μL, the flow rate was 0.8 mL/min and the column temperature was 40 °C.

For the GC/MS analysis, HCl was added to stop the whole-cell biotransformation reaction, and then 1 mM internal standard (DDA for the C_10:0_ and C_13:0_ substrates; tridecanoic acid for the C_14:0_ and C_16:0_ substrates) was added for product quantification. The reaction mixtures were extracted twice with 0.5 mL tert-butyl methyl ether. The organic phases were collected, dried with MgSO_4_ (anhydrous) and evaporated. Samples were resuspended in 40 μL of 1% trimethylchlorosilane in N,O-bis(trimethylsilyl) trifluoroacetamide and incubated at 75 °C for 30 min for derivatization. The samples were analyzed on a Shimadzu GCMS QP2010SE instrument (Tokyo, Japan) equipped with a Shimadzu SH-I-5Sil MS column (30 m × 0.25 mm × 0.25 μm, Tokyo, Japan), with helium as the carrier gas (flow rate, 0.69 mL/min; linear velocity, 30 cm/s). Mass spectra were collected using electrospray ionization. The injector and detector temperatures were set at 250 °C and 285 °C, respectively. For the analysis of the C_10:0_–C_13:0_ fatty acids, the column oven was set at 130 °C for 2 min, raised to 250 °C at a rate of 10 °C/min, held at isotherm for 3 min and then raised to 300 °C at 40 °C/min. For the C_14:0_ and C_16:0_ compounds, the temperature was maintained at 180 °C for 1 min, raised to 300 °C at 8 °C/min and held at isotherm for 5 min. Reaction products were identified by their characteristic mass fragmentation patterns [52].

### 3.8. Kinetic Analysis of P450

DDA was dissolved in DMSO and used at different concentrations (0.05–1 mM) as the substrate. The reaction was conducted with purified P450 in sodium phosphate buffer (100 mM, pH 7.5) containing 0.5 mM NADPH. The Epoch 2 microplate reader (BioTek Instruments, Inc, Winooski, VT, USA) was used to measure the absorbance value of NADPH at 340 nm and 30 °C every min [21]. The initial rate data were fitted nonlinearly with the Michaelis Menten equation to obtain the kinetic constant, and then the conversion number *k_cat_* and the catalytic efficiency *k_cat_*/*K_m_* were calculated.

### 3.9. Molecular Simulation Analysis of P450

Preparation of receptor proteins. According to the sequence and structure of the cyp153a-ncp reductase domain, protein models with a high sequence homology or similar structures in the PDB library [https://www.rcsb.org/ (accessed on 19 January 2022)] were selected as templates, and the Modeller 10.1 tool [53,54,55] was used to construct P450 reductase domain models through homologous modeling. The results are visualized in PyMOL. The MolProbity [34] [http://molprobity.biochem.duke.edu/ (accessed on 28 November 2022)] and SAVES [35] [https://saves.mbi.ucla.edu/ (accessed on 28 November 2022)] tools were used to evaluate the results, and the model with high scores was selected as the standard model of the receptor protein.

Molecular docking and analysis: The Grid tool of the AutoDockTools software was used to generate a Grid Box with an appropriate size containing all coenzymes binding pockets. The three coenzymes were successively docked by the AutoDock4 software. The configuration with the lowest binding energy and the coenzyme near the theoretical binding pocket was selected from the docking results and loaded into PyMOL software to analyze the chemical bonds between the coenzymes and the receptor protein.

## 4. Conclusions

In order to enhance the terminal hydroxylation activity of DDA catalyzed by P450, P450-directed evolution and heme synthesis enhancement were combined in this study to improve its soluble expression and enhance the electron transfer efficiency. The application of this strategy in the biosynthesis of ω-AmDDA generated an efficient whole-cell biocatalyst for PA12 monomer bioproduction. The replacement of the original P450 chimera M1 with the newly created mutants M2, with improved soluble expression, and M3, with improved expression and activity, enhanced the ω-AmDDA yields by 21.5% and 35.6%, respectively. The subsequent replacement of NAD^+^-dependent BsADH with FAD-dependent AlkJ and RBS engineering accelerated the conversion of ω-OHDDA to ω-AmDDA, which improved the ω-AmDDA yield by 136.7%. Strengthening the heme synthetic pathway led to a further 17.3% improvement in the ω-AmDDA yield. Finally, the whole-cell biocatalyst produced 9.36 mM ω-AmDDA (2.02 g/L), which was 227.0% higher than that of the original strain [6]. The HTS method established, the hot mutation sites identified in the P450 reductase domain and the strategy used to enhance the supply of heme in this work would provide helpful hints for engineering other P450-involved bioprocesses.

## Figures and Tables

**Table 1:** Positive variants obtained by directed evolution.

Clones ^a^	Amino Acid Substitutions (Synonymous Mutation)	Relative Activity ^b^
M1	G307A	1
A641	G307A/E801K (R14R/V108V/P149P/E617E/E874E)	1.32 ± 0.05
A692	G307A/I442V/N511S/G1061D (A208A/S473S/H500H)	1.35 ± 0.03
A735	G307A/T55A/K190I/K712R (R701R)	1.16 ± 0.02
A1049	G307A/ (L32L/Y459Y)	1.25 ± 0.02
A1067	G307A/V794A (E951E)	1.20 ± 0.07
B375	G307A/R14R/A18T/T371I/K494E/D629G/Q947R	1.34 ± 0.04

^a^ Mutants screened from the first round were named as “A + number”, and mutants screened from the second round were named as “B + number”. ^b^ These results were determined by the HPLC measurement of ω-OHDDA production, and all biotransformation reactions were performed in triplicate. The relative activities of the mutants obtained are given as the fold increase relative to ω-OHDDA production by M1.

**Table 2:** The kinetic constants of M1 and M3 (cyp153a-ncp^G307A/R14R/D629G^) towards DDA.

Enzyme Variant	*K_m_* (mM) ^a^	*k_cat_* (Min^−1^) ^a^	*k_cat_*/*K_m_* (Min^−1^·mM^−1^) ^a^
M1	0.27 ± 0.01	10.92 ± 0.10	40.94 ± 0.39
M3	0.21 ± 0.04	17.31 ± 1.13	81.69 ± 5.33

^a^ These results were determined by NADPH colorimetry, and the kinetic constants were obtained by fitting the initial rate data with the Michaelis–Menten equation. All biotransformation reactions were performed in triplicate.

**Table 3:** Substrate spectrum analysis of M3.

Substrate ^a^	Relative Activity ^b^
C_10:0_	1.13 ± 0.05
C_13:0_	1.65 ± 0.05
C_14:0_	1.17 ± 0.04
C_16:0_	1.93 ± 0.09

^a^ The substrate concentration used was 10 mM. ^b^ Relative activities were given as the fold increase relative to the activity of M1.

**Table 4:** Plasmids and strains used in this study.

Plasmids/Strains	Description	Source
Plasmids		
E-M1-3	pETDuet-1 derivative, *rbs3*-*cyp153a*-*ncp^G307A^* and *Bacillus cereus gdh1*; Amp^R^	[6]
C-M2-2	pCDFDuet-1 derivative, *Geobacillus stearothermophilus BsADH^C257L^*, *Chromobacterium violaceum* DSM30191 *cv2025* and *Bacillus subtilis aladh2*; Sm^R^	[6]
pET-30a(+)-M3-5	pET-30a(+) derivative, *Dactylium dendroides GOase_M3-5_*; Km^R^	[13]
pACYCDuet-1	Expression vector, p15A *ori*, double *T7lac* promoters; Cm^R^	[46]
pRSFDuet-1	Expression vector, RSF1030 *ori*, double *T7lac* promoters; Km^R^	[46]
pCDFDuet-1	Expression vector, CDF *ori*, double *T7lac* promoters; Sm^R^	[46]
E-3	pETDuet-1 derivative, *rbs3*-*cyp153a*-*ncp* and *Bacillus cereus gdh1*; Amp^R^	This study
E-M2-3	pETDuet-1 derivative, *rbs3*-*cyp153a*-*ncp^G307A/R14R^* and *Bacillus cereus gdh1*; Amp^R^	This study
E-M3-3	pETDuet-1 derivative, *rbs3*-*cyp153a*-*ncp^G307A/R14R/D629G^* and *Bacillus cereus gdh1*; Amp^R^	This study
pAC-*hemB*	pACYCDuet-1 derivative, *E. coli hemB*; Km^R^	This study
pAC-*hemE*	pACYCDuet-1 derivative, *E. coli hemE*; Sm^R^	This study
pAC-*hemH*	pACYCDuet-1 derivative, *E. coli hemH*; Sm^R^	This study
pRS-*hemB*	pRSFDuet-1 derivative, *E. coli hemB*; Km^R^	This study
pRS-*hemF*	pRSFDuet-1 derivative, *E. coli hemF*; Km^R^	This study
pAC-hemBCDE	pACYCDuet-1 derivative, *E. coli hemB*, *hemC*, *hemD* and *hemE*; Sm^R^	This study
pAC-hemEFGH	pACYCDuet-1 derivative, *E. coli hemE*, *hemF*, *hemG* and *hemH*; Sm^R^	This study
pRS-hemBCD	pRSFDuet-1 derivative, *E. coli hemB*, *hemC* and *hemD*; Km^R^	This study
pRS-hemFGH	pRSFDuet-1 derivative, *E. coli hemF*, *hemG* and *hemH*; Km^R^	This study
E-M1-3-*gdhA*	pETDuet-1 derivative, *rbs3*-*cyp153a*-*ncp^G307A^* and *E. coli gdhA*; Amp^R^	This study
E-M1-3-*icd*	pETDuet-1 derivative, *rbs3*-*cyp153a*-*ncp^G307A^* and *E. coli icd*; Amp^R^	This study
E-M1-3-*fdh1*	pETDuet-1 derivative, *rbs3*-*cyp153a*-*ncp^G307A^* and *Burkholderia stabilis 15516 fdh1*; Amp^R^	This study
pCD-*pntAB*	pCDFDuet-1 derivative, *E. coli pntAB*; Sm^R^	This study
pCD-*sthA*	pCDFDuet-1 derivative, *E. coli sthA*; Sm^R^	This study
pCD-*pntAB*-*fdh2*	pCDFDuet-1 derivative, *E. coli pntAB* and *Mycolicibacterium vaccae fdh2*; Sm^R^	This study
pCD-*sthA*-*fdh2*	pCDFDuet-1 derivative, *E. coli sthA* and *M. vaccae fdh2*; Sm^R^	This study
C-*alkJ*	pCDFDuet-1 derivative, *Pseudomonas oleovorans alkJ*, *Chromobacterium violaceum* DSM30191 *cv2025* and *Bacillus subtilis aladh2*; Sm^R^	This study
C-*rbs2*-*alkJ*	pCDFDuet-1 derivative, *rbs2-alkJ*, *Chromobacterium violaceum* DSM30191 *cv2025* and *Bacillus subtilis aladh2*; Sm^R^	This study
Strains		
B1-1	*E. coli* BL21(DE3), Δ*fadD*::*P_lacUV5_*-*alkL*	[6]
P1-1	B1-1, Δ*fadD*::*P_T7_-yaaDE*	[6]
MG1655	*E. coli* K-12 wild type	Lab stock
BL21+M3-5	B1-1 strain harboring plasmid pET-30a(+)-M3-5; Km^R^	This study
B1-1-1	B1-1 strain harboring the plasmids pAC-hemBCDE, pRS-hemFGH and E-M1-3; Sm^R^, Km^R^ and Amp^R^	This study
B1-1-2	B1-1 strain harboring the plasmids pAC-*hemB* and E-M1-3; Km^R^ and Amp^R^	This study
B1-1-3	B1-1 strain harboring the plasmids pRS-hemBCD, pAC-hemEFGH and E-M1-3; Sm^R^, Km^R^ and Amp^R^	This study
B1-1-3+Δ*yfeX*	B1-1, Δ*yfeX* strain harboring the plasmids pRS-hemBCD, pAC-hemEFGH and E-M1-3; Sm^R^, Km^R^ and Amp^R^	This study
B2-1	B1-1, T10::*P_lac_*-*hemB-T_1_*	This study
B2-2	B1-1, T10::*P_tac_*-*hemB-T_1_*	This study
B2-3	B1-1, T10::*P_T7_*-*hemB-T_1_*	This study
B3-1	P1-1 strain harboring the plasmids E-M1-3 and C*-alkJ*; Sm^R^ and Amp^R^	This study
B3-2	P1-1, Δ*yfeX*-T10::*P_tac_-hemB-T_1_* strain harboring the plasmids E-M3-3 and C-*rbs2-alkJ*; Sm^R^ and Amp^R^	This study

## Data Availability

All data needed to evaluate the conclusions in the paper are present in the paper and/or the supplementary information.

## Supplementary Materials

The following supporting information can be downloaded at: https://www.mdpi.com/article/10.3390/molecules28041758/s1, Table S1: Primers used for PCR and cloning; Figure S1: Replacement of the NADPH regeneration system; Figure S2: The homologous modeling results of the P450 reduction domain, as evaluated by MolProbity; Figure S3: The homologous modeling results of the P450 reduction domain, as evaluated by SAVES; Figure S4: Gas chromatograms for C_10:0_–C_16:0_ and their ω-hydroxylation products; Figure S5: Electron ionization mass spectral fragmentations of trimethylsiloxyl (TMS) derivatives of C_10:0_–C_16:0_ and their ω-hydroxylation products; Figure S6: Assay of active P450; Figure S7: Biotransformation of DDA to ω-AmDDA by P1-1 (E-M1-3/C-M2-2). References [6,23,24,25,26,27,34,35] are cited in the supplementary materials

## References

[1] Winnacker M. (2017). Polyamides and their functionalization: Recent concepts for their applications as biomaterials. Biomater. Sci..

[2] Carole T.M., Pellegrino J., Paster M.D. (2004). Opportunities in the industrial biobased products industry. Appl. Biochem. Biotechnol..

[3] Dachs K., Schwartz E. (1962). Pyrrolidon, capryllactam und laurinlactam als neue grundstoffe für polyamidfasern. Angew. Chem. Int. Ed..

[4] Ladkau N., Assmann M., Schrewe M., Julsing M.K., Schmid A., Buhler B. (2016). Efficient production of the Nylon 12 monomer omega-aminododecanoic acid methyl ester from renewable dodecanoic acid methyl ester with engineered *Escherichia coli*. Metab. Eng..

[5] Ahsan M.M., Jeon H., Nadarajan S., Chung T., Yoo H.W., Kim B.G., Patil M.D., Yun H. (2018). Biosynthesis of the nylon 12 monomer, ω-aminododecanoic acid with novel CYP153A, AlkJ, and ω-TA enzymes. Biotechnol. J..

[6] Ge J.W., Yang X.H., Yu H.W., Ye L.D. (2020). High-yield whole cell biosynthesis of Nylon 12 monomer with self-sufficient supply of multiple cofactors. Metab. Eng..

[7] Notonier S., Gricman L., Pleiss J., Hauer B. (2016). Semirational protein engineering of CYP153A(M.aq.)-CPRBM3 for efficient terminal hydroxylation of short- to long-chain fatty acids. ChemBioChem.

[8] Duan Y., Ba L., Gao J.W., Gao X.X., Zhu D.M., de Jong R.M., Mink D., Kaluzna I., Lin Z.L. (2016). Semi-rational engineering of cytochrome CYP153A from *Marinobacter aquaeolei* for improved omega-hydroxylation activity towards oleic acid. Appl. Microbiol. Biotechnol..

[9] Malca S.H., Scheps D., Kuhnel L., Venegas-Venegas E., Seifert A., Nestl B.M., Hauer B. (2012). Bacterial CYP153A monooxygenases for the synthesis of omega-hydroxylated fatty acids. Chem. Commun..

[10] Zhang L.L., Xie Z.Z., Liu Z.W., Zhou S.Y., Ma L.X., Liu W.D., Huang J.W., Ko T.P., Li X.Q., Hu Y.C. (2020). Structural insight into the electron transfer pathway of a self-sufficient P450 monooxygenase. Nat. Commun..

[11] Olsen M., Iverson B., Georgiou G. (2000). High-throughput screening of enzyme libraries. Curr. Opin. Biotechnol..

[12] Weissenborn M.J., Notonier S., Lang S.L., Otte K.B., Herter S., Turner N.J., Flitsch S.L., Hauer B. (2016). Whole-cell microtiter plate screening assay for terminal hydroxylation of fatty acids by P450s. Chem. Commun..

[13] Escalettes F., Turner N.J. (2008). Directed evolution of galactose oxidase: Generation of enantioselective secondary alcohol oxidases. ChemBioChem.

[14] Sevrioukova I.F., Li H.Y., Zhang H., Peterson J.A., Poulos T.L. (1999). Structure of a cytochrome P450-redox partner electron-transfer complex. Proc. Natl. Acad. Sci. USA.

[15] Hoffmann S.M., Danesh-Azari H.R., Spandolf C., Weissenborn M.J., Grogan G., Hauer B. (2016). Structure-guided redesign of CYP153A(M.aq) for the improved terminal hydroxylation of fatty acids. ChemCatChem.

[16] Green M.T. (2009). C-H bond activation in heme proteins: The role of thiolate ligation in cytochrome P450. Curr. Opin. Chem. Biol..

[17] Ge B.S., Chen Y., Yu Q., Lin X.J., Li J.Q., Qin S. (2018). Regulation of the heme biosynthetic pathway for combinational biosynthesis of phycocyanobilin in *Escherichia coli*. Process Biochem..

[18] Zhao X.R., Choi K.R., Lee S.Y. (2018). Metabolic engineering of *Escherichia coli* for secretory production of free haem. Nat. Catal..

[19] Sathesh-Prabu C., Lee S.K. (2015). Production of long-chain alpha,omega-dicarboxylic acids by engineered *Escherichia coli* from renewable fatty acids and plant oils. J. Agric. Food Chem..

[20] Munro A.W., Leys D.G., McLean K.J., Marshall K.R., Ost T.W.B., Daff S., Miles C.S., Chapman S.K., Lysek D.A., Moser C.C. (2002). P450BM3: The very model of a modern flavocytochrome. Trends Biochem. Sci..

[21] Tsotsou G.E., Cass A.E.G., Gilardi G. (2002). High throughput assay for cytochrome P450BM3 for screening libraries of substrates and combinatorial mutants. Biosens. Bioelectron..

[22] Li Q.S., Schwaneberg U., Fischer M., Schmitt J., Pleiss J., Lutz-Wahl S., Schmid R.D. (2001). Rational evolution of a medium chain-specific cytochrome P-450 BM-3 variant. Biophys. Acta Protein Struct. Molec. Enzym..

[23] Murakami K., Tsubouchi R., Fukayama M., Ogawa T., Yoshino M. (2006). Oxidative inactivation of reduced NADP-generating enzymes in *E-coli*: Iron-dependent inactivation with affinity cleavage of NADP-isocitrate dehydrogenase. Arch. Microbiol..

[24] Sakamoto N., Kotre A.M., Savageau M.A. (1975). Glutamate-dehydrogenase from *Escherichia coli*: Purification and properties. J. Bacteriol..

[25] Fukushima T., Decker R.V., Anderson W.M., Spivey H.O. (1989). Substrate channeling of NADH and binding of dehydrogenases to complex-I. J. Biol. Chem..

[26] Galkin A., Kulakova L., Tishkov V., Esaki N., Soda K. (1995). Cloning of formate dehydrogenase gene from a methanol-utilizing bacterium *Mycobacterium vaccae* N10. Appl. Microbiol. Biotechnol..

[27] Jan J., Martinez I., Wang Y.P., Bennett G.N., San K.Y. (2013). Metabolic engineering and transhydrogenase effects on NADPH availability in *Escherichia coli*. Biotechnol. Prog..

[28] Ichinose H., Hatakeyama M., Yamauchi Y. (2015). Sequence modifications and heterologous expression of eukaryotic cytochromes P450 in *Escherichia coli*. J. Biosci. Bioeng..

[29] Hausjell J., Halbwirth H., Spadiut O. (2018). Recombinant production of eukaryotic cytochrome P450s in microbial cell factories. Biosci. Rep..

[30] Reis A.C., Salis H.M. (2020). An automated model test system for systematic development and improvement of gene expression models. ACS Synth. Biol..

[31] Salis H.M., Mirsky E.A., Voigt C.A. (2009). Automated design of synthetic ribosome binding sites to control protein expression. Nat. Biotechnol..

[32] Joyce M.G., Ekanem I.S., Roitel O., Dunford A.J., Neeli R., Girvan H.M., Baker G.J., Curtis R.A., Munro A.W., Leys D. (2012). The crystal structure of the FAD/NADPH-binding domain of flavocytochrome P450 BM3. FEBS J..

[33] Wang M., Roberts D.L., Paschke R., Shea T.M., Masters B.S., Kim J.J. (1997). Three-dimensional structure of NADPH-cytochrome P450 reductase: Prototype for FMN- and FAD-containing enzymes. Proc. Natl. Acad. Sci. USA.

[34] Williams C.J., Headd J.J., Moriarty N.W., Prisant M.G., Videau L.L., Deis L.N., Verma V., Keedy D.A., Hintze B.J., Chen V.B. (2018). MolProbity: More and better reference data for improved all-atom structure validation. Protein Sci..

[35] Colovos C., Yeates T.O. (1993). Verification of protein structures: Patterns of nonbonded atomic interactions. Protein Sci..

[36] Sugishima M., Taira J., Sagara T., Nakao R., Sato H., Noguchi M., Fukuyama K., Yamamoto K., Yasunaga T., Sakamoto H. (2020). Conformational equilibrium of NADPH-cytochrome P450 oxidoreductase is essential for heme oxygenase reaction. Antioxidants.

[37] Iijima M., Ohnuki J., Sato T., Sugishima M., Takano M. (2019). Coupling of redox and structural states in cytochrome P450 reductase studied by molecular dynamics simulation. Sci. Rep..

[38] Weng H., Ding W., Shi Y., Li J., Kang Z. (2019). Enhancement of heme synthesis pathway in *Escherichia coli* via a modular optimization strategy. J. Food Sci. Biotechnol..

[39] Zhang J.L., Kang Z., Chen J., Du G.C. (2015). Optimization of the heme biosynthesis pathway for the production of 5-aminolevulinic acid in *Escherichia coli*. Sci. Rep..

[40] Zhou Y.J.J., Yang W., Wang L., Zhu Z.W., Zhang S.F., Zhao Z.B.K. (2013). Engineering NAD(+) availability for *Escherichia coli* whole-cell biocatalysis: A case study for dihydroxyacetone production. Microb. Cell Fact..

[41] Bao T., Zhang X., Zhao X.J., Rao Z.M., Yang T.W., Yang S.T. (2015). Regulation of the NADH pool and NADH/NADPH ratio redistributes acetoin and 2,3-butanediol proportion in *Bacillus subtilis*. Biotechnol. J..

[42] Sakoda H., Imanaka T. (1992). Cloning and sequencing of the gene coding for alcohol dehydrogenase of *Bacillus stearothermophilus* and rational shift of the optimum pH. J. Bacteriol..

[43] Vanbeilen J.B., Eggink G., Enequist H., Bos R., Witholt B. (1992). DNA sequence determination and functional characterization of the OCT-plasmid-encoded alkJKL genes of *Pseudomonas oleovorans*. Mol. Microbiol..

[44] Liu S., Zhang X., Liu F., Xu M.J., Yang T.W., Long M.F., Zhou J.P., Osire T., Yang S.T., Rao Z. (2019). Designing of a cofactor self-sufficient whole-cell biocatalyst system for production of 1,2-amino alcohols from epoxides. ACS Synth. Biol..

[45] Zhang Y., Sun X., Wang Q., Xu J., Dong F., Yang S., Yang J., Zhang Z., Qian Y., Chen J. (2020). Multicopy chromosomal integration using CRISPR-associated transposases. ACS Synth. Biol..

[46] Wu S.K., Zhou Y., Wang T.W., Too H.P., Wang D.I.C., Li Z. (2016). Highly regio- and enantioselective multiple oxy- and amino-functionalizations of alkenes by modular cascade biocatalysis. Nat. Commun..

[47] Gibson D.G., Young L., Chuang R.Y., Venter J.C., Hutchison C.A., Smith H.O. (2009). Enzymatic assembly of DNA molecules up to several hundred kilobases. Nat. Methods.

[48] HamediRad M., Weisberg S., Chao R., Lian J., Zhao H. (2019). Highly efficient single-pot scarless Golden Gate assembly. ACS Synth. Biol..

[49] Joo H., Arisawa A., Lin Z.L., Arnold F.H. (1999). A high-throughput digital imaging screen for the discovery and directed evolution of oxygenases. Chem. Biol..

[50] Omura T., Sato R. (1964). Carbon monoxide-binding pigment of liver microsomes. J. Biol. Chem..

[51] Panicco P., Astuti Y., Fantuzzi A., Durrant J.R., Gilardi G. (2008). P450 versus P420: Correlation between cyclic voltammetry and visible absorption spectroscopy of the immobilized heme domain of cytochrome P450 BM3. J. Phys. Chem. B.

[52] Rontani J.F., Aubert C. (2004). Trimethylsilyl transfer during electron ionization mass spectral fragmentation of some omega-hydroxycarboxylic and omega-dicarboxylic acid trimethylsilyl derivatives and the effect of chain length. Rapid Commun. Mass Spectrom..

[53] Webb B., Sali A. (2016). Comparative protein structure modeling using MODELLER. Curr. Protoc. Bioinf..

[54] Webb B., Sali A. (2021). Protein structure modeling with MODELLER. Methods Mol. Biol..

[55] Sali A., Blundell T.L. (1993). Comparative protein modelling by satisfaction of spatial restraints. J. Mol. Biol..

