# Supplementary material for: Improved Bioproduction of the Nylon 12 Monomer by Combining the Directed Evolution of P450 and Enhancing Heme Synthesis
Source: Molecules. 2023 Feb 13;28(4):1758. doi: 10.3390/molecules28041758 (PMC9963201; doi:10.3390/molecules28041758)
Supplement: Supplementary file 1 [file molecules-28-01758-s001.zip › molecules-2198604-supplementary.pdf]

Supporting information for

Article

Improved Bioproduction of the Nylon 12 Monomer by Combining the Directed Evolution of P450 and Enhancing Heme Synthesis

Jiaming Yu [1,2], Jiawei Ge[2], Hongwei Yu [1,2,*] and Lidan Ye [1,2,*]

1 Key Laboratory of Biomass Chemical Engineering (Education Ministry), College of Chemical and Biological Engineering, Zhejiang University, Hangzhou 310058, China
2 Institute of Bioengineering, College of Chemical and Biological Engineering, Zhejiang University, Hangzhou 310058, China
* Correspondence: yuhongwei@zju.edu.cn (H.Y.); yelidan@zju.edu.cn (L.Y.)

Contents:

Supporting Tables **S1**
Table S1. Primers used for PCR and cloning S2

Supporting Figures **S5**
Figure S1. Replacement of the NADPH regeneration system S5
Figure S2. The homologous modeling results of P450 reduction domain evaluated by MolProbity S5
Figure S3. The homologous modeling results of P450 reduction domain evaluated by SAVES S6
Figure S4. Gas chromatograms for $C_{10:0}$-$C_{16:0}$ and their ω-hydroxylation products S7
Figure S5. Electron ionization mass spectral fragmentations of trimethylsiloxyl (TMS) derivatives of $C_{10:0}$-$C_{16:0}$ and their ω-hydroxylation products S8
Figure S6. Assay of active P450 .... S9
Figure S7. Biotransformation of DDA to ω-AmDDA by P1-1 (E-M1-3/C-M2-2) S9

References **S10**

Supporting Tables

Table S1. Primers used for PCR and cloning

Primer name	Primer sequence (5′-3′) [a]	Description
CYPCPR-Gibson-F	TAATTTTGTTTAACTTTAACAGGAGGAACGGCATGCCGACTTTACCGCGTA	To construct P450 mutants
CYPCPR-Gibson-R	TCCAGATCGCTGTAACCCATTATATATCTCCTTAGAATTCTTACCCAGCCCACAC	
Petduet-Gibson-F	ATGGGTTACAGCGATCTGGA	
Petduet-Gibson-R	AAAGTTAAACAAAATTATTTCTAGAGGGGAATTG	
TP450-BsaI-GCCA-F	GGCTACGGTCTCAGCCATATGCCGACTTTACCGCGTAC	To construct His-Tagged P450
TP450-BsaI-TACC-R	GGCTACGGTCTCACCATGCCGTTCCTCCTGTTAAAGT	
NHis-BsaI-F	GGCTACGGTCTCCATGGGCAGCAGCCATCATCATCATCATCACAGCAGCGGCCTGGTGCCGCGCGGCAGCCATGAGACCGTAGCC	
NHis-BsaI-R	GGCTACGGTCTCATGGCTGCCGCGCGGCACCAGGCCGCTGCTGTGATGATGATGATGATGGCTGCTGCCCATGGAGACCGTAGCC	
TP450-BsaI-ACTG-F	GGCTACGGTCTCCACTGAGAATTCTAAGGAGATATATAATGGGT	
TP450-BsaI-AGGC-R	GGCTACGGTCTCACGGACCCAGCCCACACGTCTTTTG	
CHis-BsaI-F	GGCTACGGTCTCCTCCGTCGACAAGCTTGCGGCCGCACTCGAGCACCACCACCACCACCACTGAGAGACCGTAGCC	
CHis-BsaI-R	GGCTACGGTCTCTCAGTGGTGGTGGTGGTGGTGCTCGAGTGCGGCCGCAAGCTTGTCGACGGAGGAGACCGTAGCC	
hemB-1-F	CTTTAATAAGGAGATATACCATGACAGACTTAATCCAACGCCC	To construct plasmid pAC-hemBCDE
hemB-1-R	TCTGCAGGCGCGCCGAGCTCTTAACGCAGAATCTTCTTCTCAGCC	
hemB-2-F	GTATAAGAAGGAGATATACATATGACAGACTTAATCCAACGCCC	To construct plasmid pAC-*hemB*
hemB-2-R	GTTTCTTTACCAGACTCGAGTTAACGCAGAATCTTCTTCTCAGCC	
hemB-3-R	ACCTGCAGGCGCGCCGAGCTCTTAACGCAGAATCTTCTTCTCAGCC	To construct plasmid pRS-*hemB*
hemC-1-F	AGAGCTCGGCGCGCCTGCAGATGTTAGACAATGTTTTAAGAATTGCCACACG	To construct plasmid pAC-hemBCDE
hemC-1-R	ACTCATATATTTTCCTCCTTTCATGCCGGGGCGTCTCCGTTATA	
hemC-2-F	TATAAGAAGGAGATATACATATGTTAGACAATGTTTTAAGAATTGCC	To construct plasmid pRS-hemBCD
hemC-2-R	TTTCCTCCTTTTATAGATCTTCATGCCGGGGCGTCTCCGTTA	

hemD-1-F	GCATGAAAGGAGGAAAATATATGAGTATCCTTGTCACCCGCC	To construct plasmid pAC-hemBCDE
hemD-1-R	CATTATGCGGCCGCAAGCTTTTATTGTAATGCCCGTAAAAGCGC	
hemD-2-F	AGATCTATAAAAGGAGGAAAATATATGAGTATCCTTGTCACCCGCC	To construct plasmid pRS-hemBCD
hemD-2-R	GTTTCTTTACCAGACTCGAGTTATTGTAATGCCCGTAAAAGCGC	
hemE-1-F	TATAAGAAGGAGATATACATATGACCGAACTTAAAAACGATCGTTATCT	To construct plasmid pAC-*hemE*
hemE-1-R	GTTTCTTTACCAGACTCGAGTTAGCGGTGATACTGTTCAGACAGT	
hemE-2-F	CTTTAATAAGGAGATATACCATGACCGAACTTAAAAACGATCGTTATCT	To construct plasmid pAC-hemEFGH
hemE-2-R	GAGCTCTTAGCGGTGATACTGTTCAGACAGT	
hemF-1-F	CTTTAATAAGGAGATATACCATGAAACCCGACGCACACCA	To construct plasmid pRS-*hemF*
hemF-1-R	CATTATGCGGCCGCAAGCTTTACACCCAATCCCTGACCTT	
hemF-2-F	AGTATCACCGCTAAGAGCTCATAAAAGGAGGAAAATATATGAAACCCGACGCACACCA	To construct plasmid pAC-hemEFGH
hemF-2-R	TTTTATCTGCAGTTACACCCAATCCCTGACCTT	
hemG-1-F	TATAAGAAGGAGATATACATATGAAAACATTAATTCTTTTCTCAACAAGGG	To construct plasmid pRS-hemFGH
hemG-1-R	ACGCATATATTTTCCTCCTTTTATTTCAGCGTCGGTTTGTCG	
hemG-2-F	ATTGGGTGTAACTGCAGATAAAAGGAGGAAAATATATGAAAACATTAATTCTTTTCTC	To construct plasmid pAC-hemEFGH
hemG-2-R	CTTAAGCATTATGCGGCCGCTTATTTCAGCGTCGGTTTGTCG	
hemH-1-F	AAATAAAAGGAGGAAAATATATGCGTCAGACTAAAACCGGTATC	To construct plasmid pRS-hemFGH
hemH-1-R	GTTTCTTTACCAGACTCGAGTTAGCGATACGCGGCAACAA	
hemH-2-F	TATAAGAAGGAGATATACATATGCGTCAGACTAAAACCGGTATC	To construct plasmid pAC-*hemH*
pACYC-1-F	AAGCTTGCGGCCGCATAATG	To construct plasmids pAC-*hemE* and pRS-*hemF*
pACYC-1-R	GGTATATCTCCTTATTAAAGTTAAACAAAATTATTTCTACAGGG	
pACYC-2-F	CTCGAGTCTGGTAAAGAAACCGC	To construct plasmids pAC-*hemB*, pAC-*hemE*, pAC-*hemH* pRS-hemBCD and pRS-hemFGH
pACYC-2-R	ATGTATATCTCCTTCTTATACTTAACTAATATACTAAGATGGGG	
pACYC-3-F	GAGCTCGGCGCGCCTGCAGG	To construct plasmids pRS-*hemB* and pRS-*hemF*
pACYC-3-R	GGTATATCTCCTTATTAAAGT	

pACYC-4-F	GCGGCCGCATAATGCTTAAG	To construct plasmid pAC-hemEFGH
gdhA-EcoRI-RBS-F	NNNNGAATTCTAAGGAGATATATAATGGATCAGACATATTCTCTGGAG	To construct plasmid pETDuet-1-*rbs3-cyp153a-ncp*^{G307A}-*gdhA* (E-M1-3-*gdhA*)
gdhA-KpnI-R	NNNNGGTACCTTAAATCACACCCTGCGCCA	
icd-EcoRI-RBS-F	NNNNGAATTCTAAGGAGATATATAATGGAAAGTAAAGTAGTTGTTCCGG	To construct plasmid pETDuet-1-*rbs3-cyp153a-ncp*^{G307A}-*icd* (E-M1-3-*icd*)
icd-KpnI-R	NNNNGGTACCTTACATGTTTTCGATGATCGCG	
fdh-EcoRI-RBS-F	NNNNGAATTCTAAGGAGATATATAATGGCCACCGTGCTGTGTG	To construct plasmid pETDuet-1-*rbs3-cyp153a-ncp*^{G307A}-*fdh1* (E-M1-3-*fdh1*)
fdh-KpnI-R	NNNNGGTACCTTAGGTTAAGCGATAGCTCTGGG	
pntAB-NcoI-F	NNNNCCATGGGCCGAATTGGCATACCAAGAGAACG	To construct plasmid pCD-*pntAB*
pntAB-SacI-R	NNNNGAGCTCTTACAGAGCTTTCAGGATTGCAT	
sthA-NcoI-F	NNNNCCATGGGCCCACATTCCTACGATTACGATGCCA	To construct plasmid pCD-*sthA*
sthA-SacI-R	NNNNGAGCTCTTAAAACAGGCGGTTTAAACCGTTTAACG	
fdh2-BglII-F	NNNNAGATCTATGGCCAAAGTGCTGTGTGT	To construct plasmids pCD-*pntAB-fdh2* and pCD-*sthA-fdh2*
fdh2-BamHI-R	NNNNGGATCCTTACACGGCTTTTTAAATTTGGCTG	
pCDFDuet-BamHI-F	NNNNGGATCCCTCGAGTCTGGTAAAGAAAC	
pCDFDuet-BglII-R	NNNNAGATCTGCATGTATATCTCCTTCTTA	
alkJ-F	ATGTACGACTATATAATCGTTGGTGCTGGA	To construct plasmid C-*alkJ*
alkJ-R	TTACATGCAGACAGCTATCATGGC	
C-M2-2-F	TGATAGCTGTCTGCATGTAATCTGGTAAAGAAACCGCTGC	
C-M2-2-R	ACGATTATATAGTCGTACATCGGATCCTGGCTGTGGTGAT	
rbs2-F	GGCTACGGTCTCATTCCTCAATAGCCTTGACTAAGGAGGTAACTATGGGGAGACCGTAGCC	To construct plasmid C-*rbs2-alkJ*
rbs2-R	GGCTACGGTCTCCCCATAGTTACCTCCTTAGTCAAGGCTATTGAGGAATGAGACCGTAGCC	
C-alkJ-BsaI-ATGG-F	GGCTACGGTCTCCATGGGCAGCAGCCATCACCATCATCACCACAGCCAGGATCCGATGTACGACTA	
C-alkJ-BsaI-TTCC-R	GGCTACGGTCTCGGGAATTGTTATCCGCTCACAATTCCCCTATAGTGAGTCGTATTAATTTCGA	

[a] Underline formatting indicates restriction sites.

Supporting Figures

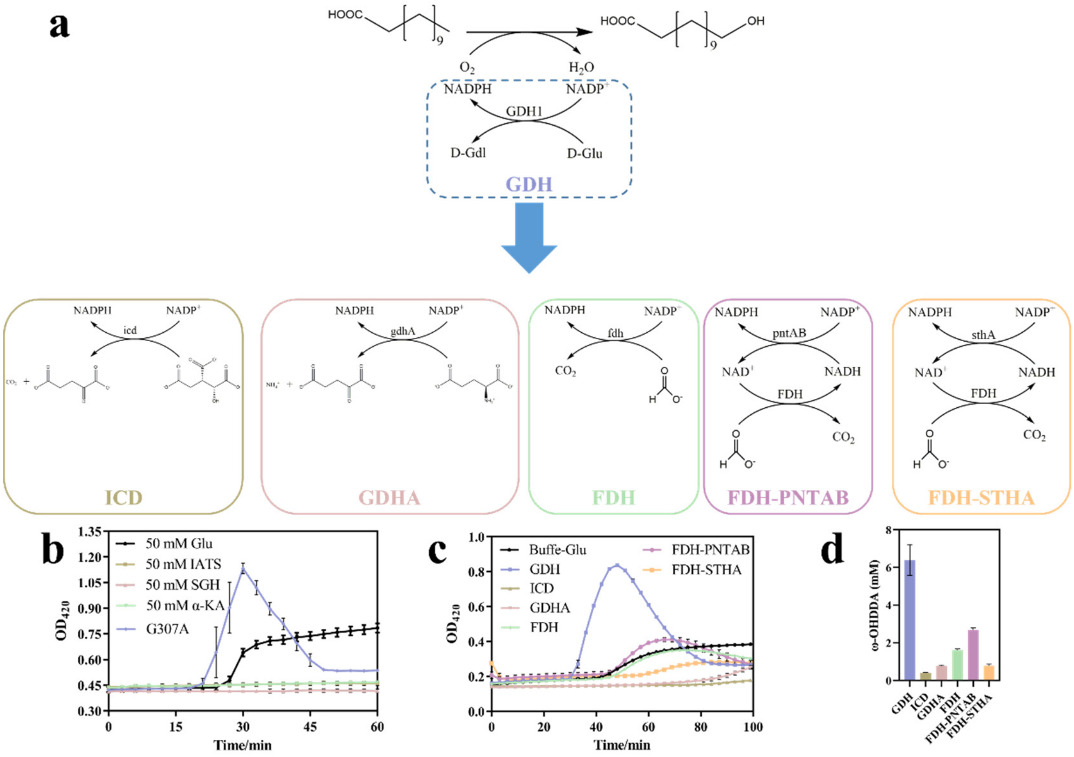

Figure S1. Replacement of the NADPH regeneration system. (**a**) Schematic of the NADPH regeneration systems tested. From left to right are ICD [1], GDHA [2], FDH [3], FDH-PNTAB [4,5] and FDH-STHA [4,5] systems. (**b**) The substrates and by-products (Glu, IATS, SGH and α-KA) required by NADPH regeneration system were detected by ABTS colorimetry. IATS, isocitric acid trisodium salt (substrate of ICD); SGH, sodium glutamate hydrate (substrate of GDHA); α-KA, 2-ketoglutaric acid (product of ICD); G307A, reaction solution after whole-cell biotransformation of M1. (**c**) ω-OHDDA production using different NADPH regeneration systems as detected by ABTS colorimetry. (**d**) ω-OHDDA production using different NADPH regeneration systems as detected by HPLC.

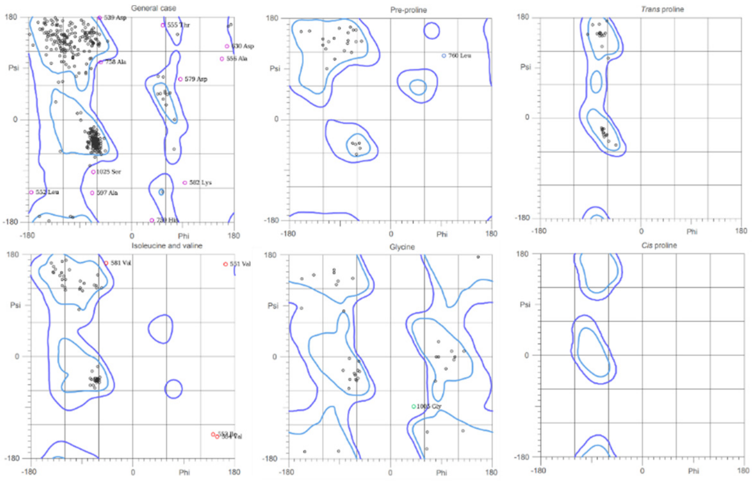

Figure S2. The homologous modeling results of P450 reduction domain evaluated by MolProbity [6].

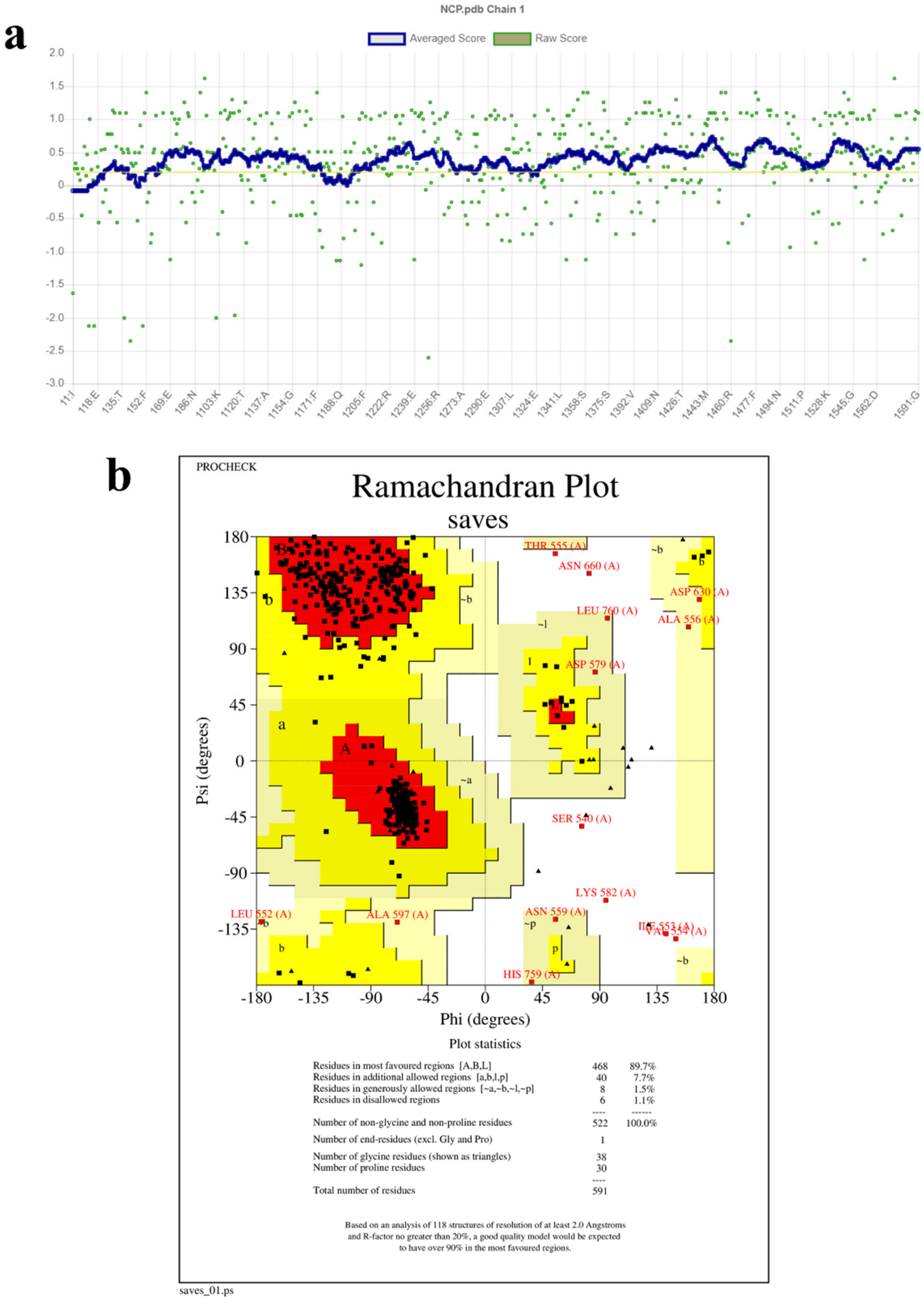

Figure S3. The homologous modeling results of P450 reduction domain evaluated by SAVES [7]. (**a**) VERIFY3D module evaluation results, which are used to test the three-dimensional structure compatibility of the protein model and at least 80% of the amino acids need to have scored >= 0.2 in the 3D/1D profile. (**b**) Ramachandran plot evaluation results.

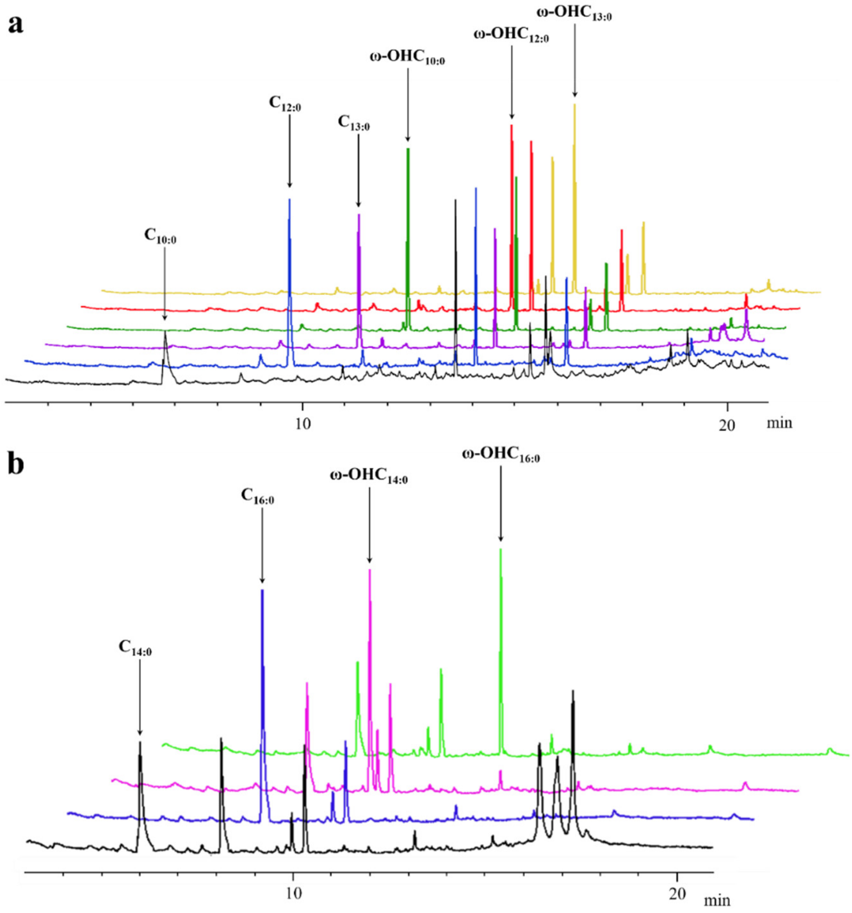

Figure S4. Gas chromatograms for $C_{10:0}$-$C_{16:0}$ and their ω-hydroxylation products. Abbreviations: $C_{10:0}$, decanoic acid; $C_{12:0}$, lauric acid; $C_{13:0}$, tridecanoic acid; C14:0, myristic acid; C16:0, palmitic acid; ω-$OHC_{10:0}$, 10-hydroxydecanoic acid; ω-$OHC_{12:0}$, 12-hydroxylauric acid; ω-$OHC_{13:0}$, 13-hydroxytridecanoic acid; ω-$OHC_{14:0}$, 14-hydroxymyristic acid; ω-$OHC_{16:0}$, 16-hydroxypalmitic acid.

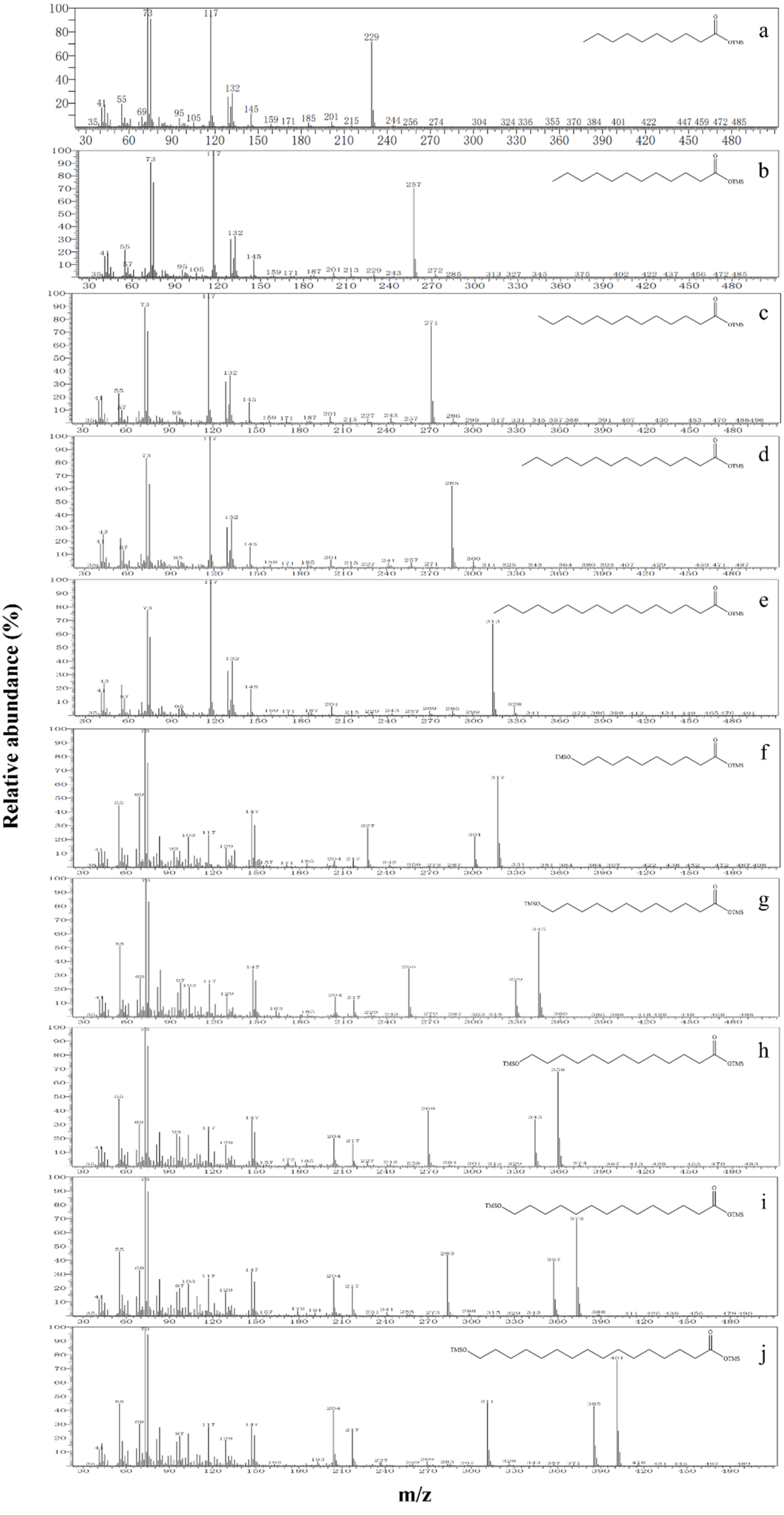

Figure S5. Electron ionization mass spectral fragmentations of trimethylsiloxyl (TMS) derivatives of $C_{10:0}$-$C_{16:0}$ and their ω-hydroxylation products. The compounds were identified as decanoic acid (**a**), lauric acid

(**b**), tridecanoic acid (**c**), myristic acid (**d**), palmitic acid (**e**), 10-hydroxydecanoic acid (**f**), 12-hydroxylauric acid (**g**), 13-hydroxytridecanoic acid (**h**), 14-hydroxymyristic acid (**i**), and 16-hydroxypalmitic acid (**j**). The common fragment ion at m/z = 73 occurs upon the loss of one TM ester group. Characteristic peaks of each fatty acids and ω-hydroxycarboxylic acid derivative are indicated in the corresponding mass spectra.

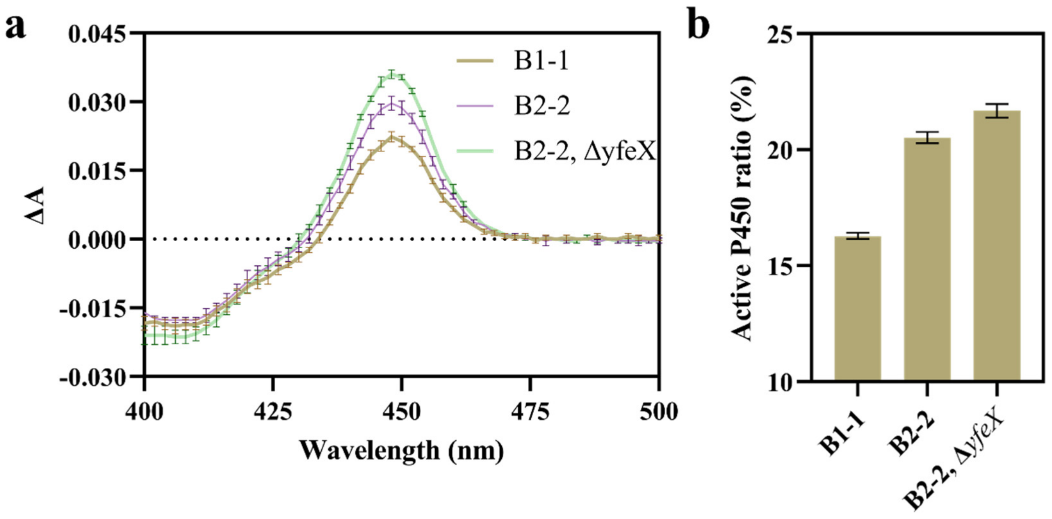

Figure S6. Assay of active P450. (**a**) CO spectra of P450. The absorbance at 490 nm was used as the "0" scale, i.e. $\Delta A = A_n - A_{490}$. (**b**) Content of active P450. The content of total P450 was determined by BCA kit [Sangon Biotech Ltd. (Songjiang District, Shanghai, China)].

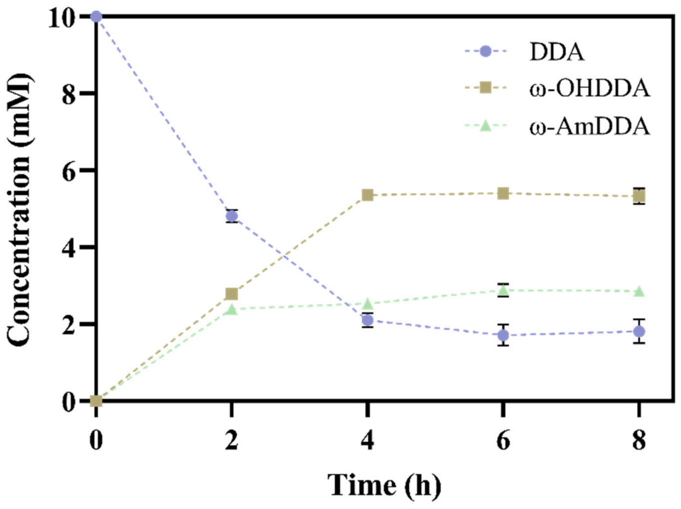

Figure S7. Biotransformation of DDA to ω-AmDDA by P1-1 (E-M1-3/C-M2-2) [8]. The reaction was performed with 50 g cww/L of resting cells in sodium phosphate buffer (100 mM, pH 8.0) containing 1% (w/v) D-glucose, $NH_3{\cdot}H_2O/NH_4Cl$ (200 mM, $NH_3{\cdot}H_2O:NH_4Cl$ = 1:10) and 10.0 mM DDA (2% DMSO). Temperature was maintained at 30°C, agitation speed was maintained at 220 rpm, pH was maintained at 7.5-8.0 and the concentration of D-glucose was maintained at 0.5-1% (w/v) throughout the biotransformation process. All biotransformation reactions were performed in triplicate, and error bars represent standard deviations.

References

1. Murakami, K.; Tsubouchi, R.; Fukayama, M.; Ogawa, T.; Yoshino, M. Oxidative inactivation of reduced NADP-generating enzymes in *E-coli*: Iron-dependent inactivation with affinity cleavage of NADP-isocitrate dehydrogenase. *Arch. Microbiol.* **2006**, *186*, 385-392.
2. Sakamoto, N.; Kotre, A.M.; Savageau, M.A. Glutamate-dehydrogenase from *Escherichia coli*: Purification and properties. *J. Bacteriol.* **1975**, *124*, 775-783.
3. Fukushima, T.; Decker, R.V.; Anderson, W.M.; Spivey, H.O. Substrate channeling of NADH and binding of dehydrogenases to complex-I. *J. Biol. Chem.* **1989**, *264*, 16483-16488.
4. Galkin, A.; Kulakova, L.; Tishkov, V.; Esaki, N.; Soda, K. Cloning of formate dehydrogenase gene from a methanol-utilizing bacterium *Mycobacterium vaccae* N10. *Appl. Microbiol. Biotechnol.* **1995**, *44*, 479-483.
5. Jan, J.; Martinez, I.; Wang, Y.P.; Bennett, G.N.; San, K.Y. Metabolic engineering and transhydrogenase effects on NADPH availability in *Escherichia coli*. *Biotechnol. Prog.* **2013**, *29*, 1124-1130.
6. Williams, C.J.; Headd, J.J.; Moriarty, N.W.; Prisant, M.G.; Videau, L.L.; Deis, L.N.; Verma, V.; Keedy, D.A.; Hintze, B.J.; Chen, V.B.; et al. MolProbity: More and better reference data for improved all-atom structure validation. *Protein Sci.* **2018**, *27*, 293-315.
7. Colovos, C.; Yeates, T.O. Verification of protein structures: Patterns of nonbonded atomic interactions. *Protein Sci.* **1993**, *2*, 1511-1519.
8. Ge, J.W.; Yang, X.H.; Yu, H.W.; Ye, L.D. High-yield whole cell biosynthesis of Nylon 12 monomer with self-sufficient supply of multiple cofactors. *Metab. Eng.* **2020**, *62*, 172-185.
